# Large benefits to youth-focused HIV treatment-as-prevention efforts in generalized heterosexual populations: An agent-based simulation model

**DOI:** 10.1371/journal.pcbi.1007561

**Published:** 2019-12-17

**Authors:** John E. Mittler, James T. Murphy, Sarah E. Stansfield, Kathryn Peebles, Geoffrey S. Gottlieb, Neil F. Abernethy, Molly C. Reid, Steven M. Goodreau, Joshua T. Herbeck

**Affiliations:** 1 Department of Microbiology, University of Washington, Seattle, WA, United States of America; 2 Department of Anthropology, University of Washington, Seattle, WA, United States of America; 3 Department of Epidemiology, University of Washington, Seattle, WA, United States of America; 4 Department of Medicine, University of Washington, Seattle, WA, United States of America; 5 Department of Global Health, University of Washington, Seattle, WA, United States of America; 6 Department of Biomedical Informatics and Medical Education, University of Washington, Seattle, WA, United States of America; 7 Department of Health Services, University of Washington, Seattle, WA, United States of America; 8 Center for Studies in Demography and Ecology, University of Washington, Seattle, WA, United States of America; ETH Zurich, SWITZERLAND

## Abstract

Predominantly heterosexual HIV-1 epidemics like those in sub-Saharan Africa continue to have high HIV incidence in young people. We used a stochastic, agent-based model for age-disparate networks to test the hypothesis that focusing uptake and retention of ART among youth could enhance the efficiency of treatment as prevention (TasP) campaigns. We used the model to identify strategies that reduce incidence to negligible levels (i.e., < 0.1 cases/100 person-years) 20–25 years after initiation of a targeted TasP campaign. The model was parameterized using behavioral, demographic, and clinical data from published papers and national reports. To keep a focus on the underlying age effects we model a generalized heterosexual population with average risks (i.e., no MSM, no PWIDs, no sex workers) and no entry of HIV+ people from other regions. The model assumes that most people (default 95%, range in variant simulations 60–95%) are “linkable”; i.e., could get linked to effective care given sufficient resources. To simplify the accounting, we assume a rapid jump in the number of people receiving treatment at the start of the TasP campaign, followed by a 2% annual increase that continues until all linkable HIV+ people have been treated. Under historical scenarios of CD4-based targeted ART allocation and current policies of untargeted (random) ART allocation, our model predicts that viral replication would need to be suppressed in 60–85% of infected people at the start of the TasP campaign to drive incidence to negligible levels. Under age-based strategies, by contrast, this percentage dropped by 18–54%, depending on the strength of the epidemic and the age target. For our baseline model, targeting those under age 30 halved the number of people who need to be treated. Age-based targeting also minimized total and time-discounted AIDS deaths over 25 years. Age-based targeting yielded benefits without being highly exclusive; in a model in which 60% of infected people were treated, ~87% and ~58% of those initiating therapy during a campaign targeting those <25 and <30 years, respectively, fell outside the target group. Sensitivity analyses revealed that youth-focused TasP is beneficial due to age-related risk factors (e.g. shorter relationship durations), and an age-specific herd immunity (ASHI) effect that protects uninfected adolescents entering the sexually active population. As testing rates increase in response to UNAIDS 90-90-90 goals, efforts to link all young people to care and treatment could contribute enormously to ending the HIV epidemic.

## Author summary

We used an agent-based model for heterosexual epidemics to evaluate the effects of focusing treatment-as-prevention (TasP) efforts on young people. To keep the focus on age effects, we modeled populations with average risks (i.e., no MSM, no PWIDs, no sex workers). For our baseline model, we found that a long-term TasP program that focuses on HIV+ people under the age of 30 can halve the number of people who need to be treated in order to drive incidence to zero. Within the context of an overall expansion of care, we were able to identify multiple scenarios in which a successful youth-focused TasP campaign also expanded care for older people. Interestingly, we continued to see strong advantages to age-based TasP in sensitivity tests in which we removed key age-related risks (e.g., the tendency for young people to have shorter relationships). Youth-focused TasP succeeds, in large part, because it protects HIV- adolescents entering the sexually active population; i.e., a population that would otherwise be at high risk not only for getting infected but also for spreading the virus to others. This protection of adolescents leads to a steadily expanding AIDS-free generation that drives the virus to extinction.

## Introduction

Despite the scale-up of antiretroviral treatment, HIV continues to be a major source of mortality in people ages 15–24 in low- and middle-income (LMIC) countries [1]. This can be traced to high rates of infection [2], low testing rates [3], difficulties linking young people to care [4], and low treatment adherence rates compared to older people [5]. HIV infection is distributed unevenly between the sexes at these ages, with young women having 3- to 5-fold higher rates of infection than young men [2, 6–10]. Higher rates of infection in young women are in part due to higher per-act probabilities of infection [11] and age-disparate relationships with older men [12–14] who are more likely to be HIV positive and less likely to use condoms [15–18]. These gaps in coverage have led to numerous calls to ramp-up testing and treatment of young people [19–20].

Two computational models suggest that youth-focused treatment as prevention (TasP) could reduce HIV incidence and/or increase quality-adjusted life years (QALYs) [21, 22]. These models assume that young people are more likely than older people to have short-term relationships [22–26]. They also include, either implicitly [21] or explicitly [22], the potential for treatment of young people to protect adolescents entering the sexually active population. Neither of these studies, however, makes a particularly strong case for youth-focused TasP. Alsallaq *et al*. [21] show a small increase in cost-effectiveness of youth-focused treatment parameterized for a specific country (Kenya) and a specific set of cost values. Bershteyn *et al*. [22] propose a slightly more general model that predicts stronger advantages to age-based TasP; however, they conclude with the pessimistic note that age-targeted TasP is "unlikely to eliminate HIV epidemics.” These models, however, do not account for two age-related risks: age-related declines in coital frequency within relationships [27–30] and higher per-act rates of infection in young people [31]. Also, since people infected with high setpoint viral load (SPVLs) viruses (i.e., more virulent viruses) die earlier in the absence of therapy and since older HIV+ people will, on average, have been infected for a longer time, older HIV+ people may be infected with fewer high SPVL viruses before the start of a TasP campaign. This opens up the possibility that youth-focused TasP, by treating individuals infected with higher SPVLs, could select for lower SPVL viruses, a factor that could reduce incidence and HIV-related mortality over time.

Factors that increase risk to youth need to be weighed against factors that protect youth, namely higher rates of condom usage [15–18] and slower progression to AIDS in the absence of therapy compared to older people [32]. Also, the tendency for youth not to have been infected a long time means that youth-focused treatment may treat fewer people who are in the AIDS stage of infection, though this will be offset by a slightly greater number who are in the acute phase of infection.

Weighing the importance of these age-related risks and benefits is difficult because they occur in the context of a demographic network with age-dependent relationship durations and complex patterns of age-related homophily (i.e., the tendency of people to partner with people their own age) in which women also tend to partner with older men. Under these conditions, network models predict that the mean degree of the population will depend on the age distribution–a distribution that can, in turn, be profoundly influenced by HIV-related mortality.

To address these complexities, we used an agent-based model for HIV epidemiology that accounts for SPVL variation to test the long-term effects of targeting young people for linkage to effective care in a generalized heterosexual epidemic. To keep the focus on age effects, we model a population with risk behaviors characteristic of the general population; that is, we do not explicitly model high-risk groups such as men who have sex with men (MSM), sex workers, and people who inject drugs (PWIDs). The model, which utilizes established routines for the underlying social network dynamics, was parameterized to reproduce broad features of sub-Saharan HIV-1 epidemics. To create a basis for comparison, we compared age-based targeting strategies to untargeted treatment (similar to currently recommended Test and Treat) and to targeting strategies based on CD4 count (historically recommended by the UNAIDS/WHO), viral load, and combinations of CD4 and age. Because of age-disparate relationships, we also tested strategies with sex-specific age targets, hypothesizing that a higher age target for men could improve age-based TasP by protecting younger female partners.

For any given strategy and coverage level (i.e., percent of HIV+ people who are virally suppressed after the TasP campaign), we quantified total and time-discounted AIDS deaths over 25 years, person-years of therapy over 25 years, and incidence 20–25 years after the start of the TasP campaign. We also measured the percentage of people who receive therapy specifically as a result of being a member of a target group (i.e., the relative inclusivity/exclusivity of the strategy). Finally, we performed sensitivity analyses in which we removed each of the age-related risk factors and altered parameters for testing rates, dropout rates, background incidence, and the percentage of people who could potentially be linked to care under a vigorous TasP campaign.

## Methods

### Software package

We used the ***Evonet_HIV*** package (https://github.com/EvoNetHIV), a stochastic, agent-based HIV epidemic model that accounts for a broad set of virological, immunological, behavioral, and epidemiological phenomena [33–35]. Each agent has attributes such as age, sex, HIV status, viral load, CD4 count (discretized into 5 bins), and HIV diagnosis status. Partnership lists and agent attributes (such as viral load and CD4 counts) were updated sequentially each day. The formation and dissolution of sexual partnerships were modeled using separable temporal exponential random graph model (STERGM) terms [36]. Since the model accounts for changes in behavior with age (e.g., young people having shorter relationships and preferentially forming attachments with other young people) and infection status (e.g., AIDS patients dying and/or having lower probabilities of sex) the model can be classified as a time-evolving adaptive network. The R package includes modules for HIV testing; viral load changes within hosts; age-dependent relationship durations; age- and viral load-dependent CD4 decline; CD4-dependent probabilities of sex; the effect of treatment on viral load; age- and diagnosis-dependent condom use; and age-, sex-, condom-, circumcision-, and viral load-dependent transmission rates. Different modules (sexual relational formation, viral load updates, etc.) were updated sequentially each time step.

Within-host viral dynamics, CD4 progression patterns, probabilities of dying of AIDS, testing procedures, and transmission probabilities were, for the most part, identical to those in previous studies using ***Evonet_HIV*** [33–35]. In the sections below, we summarize features that differ from these previous studies and/or that relate specifically to age (i.e., the key assumptions of this study). Additional details about the software are given in S1 Supplementary Methods and at github.com/EvoNetHIV.

### Data sources

We have derived most of the parameters from studies of sub-Saharan countries (mainly South Africa); however, we have used parameters from other regions when they were of significantly better quality and/or more applicable to our model. We give more details about data sources, algorithms, and network estimation procedures in the Supplemental Methods.

### Age-specific relationship durations

To approximate the tendency of young people to have short-term relationships [22–26], we divided the population into two groups: agents in group 1 (primarily composed of young people) tend to form short-term partnerships (default 2 years), agents in group 2 (primarily composed older people) tend to form long-term partnerships (default 10 years). We assumed that 90% of agents entering the sexually active population at age 16 belong to group 1. Agents in group 1 (median age 27) were assumed to have a small probability (default 0.00011 per day = ~5.5% per year) of transitioning to group 2 (median age 44). That is, agents exhibit a tendency to have longer partnerships as they age. These values were chosen so that the mean duration for people under 25 will approximate the midpoint of estimates in refs [23, 37, 38] and so that the ratio of the number partners per unit time for people under 25 to those over 40 is consistent with refs 23–26 (however, see below for caveats). Relationships between people from different groups were assumed to break up each day with probability 1/(365*AveDur), where *AveDur* is the geometric mean of each partner’s duration tendency. Use of the geometric mean (i.e., the square root of the product) means that relationship durations are determined more strongly by the partner with the shorter relationship tendency. With the parameters given above, relationships will last an average of ~6 years in a typical simulation, with ~40% and ~60% of agents, respectively, belonging to groups 1 and 2.

We are aware that our age-specific relationship terms provide but a loose approximation to a broad, but heterogeneous (and potentially unreliable) swath of data. Nguyen *et al*. [37], for example, show that estimates can vary 3-fold depending on methods used to analyze the data. Fortunately, we are able to show below that our main results do not depend on the exact values. Rather than trying to create a more complex model or trying to fit of this model to additional data, we have elected to stick with these approximate values and then examine the effect of changing the relationship duration terms in sensitivity tests (including tests in relationship durations are assumed to be completely independent of age).

### Age- and sex-specific relationship formation terms

**Fig 1:**
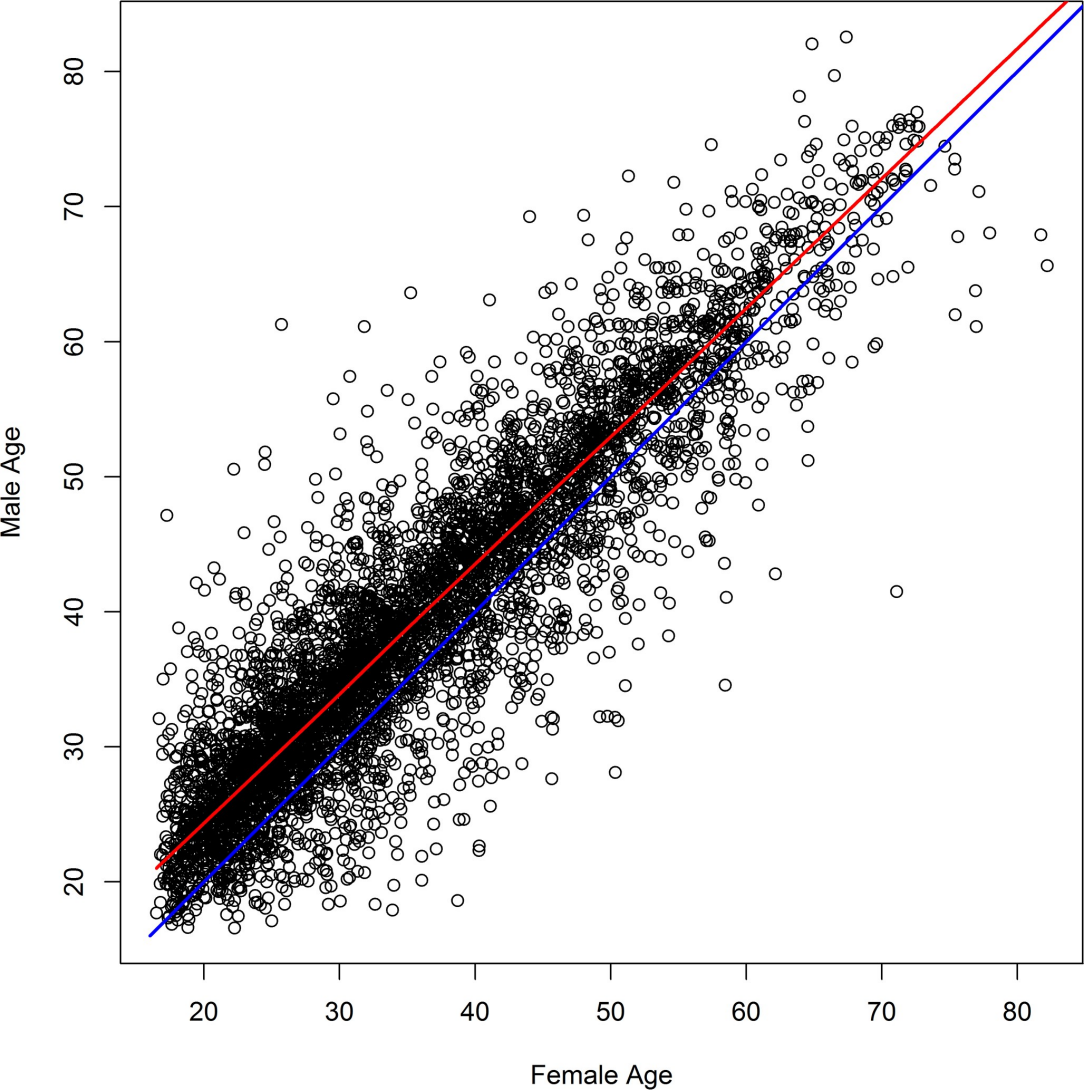
Ages of sexually active males plotted against the age of their sexually active female partners. While the model allows for partnerships between people of different ages, the majority of people partner with someone who is about the same age after accounting for age-disparate relationships. The red line is a lowess fit to the data. The blue line is the *y* = *x* line that would occur if women partnered only with men who were exactly the same age. This graph shows data from a simulation in which treatment had reduced incidence to zero.

To represent age-disparate relationships, we added a network term that pushes the average age difference between men and women to equal a specified value (default: women partner with men who are, on average, 4 years older [12, 13, 39]). To mimic the tendency of people to partner with others who are about the same age, we added an additional term that forces the average age difference in the population (after adjusting for the average male-female age difference) to equal a specified value (default 4 years). With these default values, 20-year-old women will typically partner with men between 20 and 28. In Fig 1, we give an example age-age plot from one of our simulations.

To increase prevalence in young women relative to young men [26], we also added network terms for the sex-specific cross-sectional prevalence of relational concurrency [40, 41] (defaults: Men 0.24, Women 0.04).

### Age-dependent probabilities of coitus

Following data in refs [25–28], we modeled declining probabilities of coitus, *P*_sex_, with age as follows:

$$P_{sex}=0,AveAge<16,$$

$$P_{sex}=P_{sex19}*\left[1–\left(AveAge–19\right)/\left(75–19\right)\right],$$

$$P_{sex}=0,AveAge>75,$$

where *AveAge* is the average age of the partners and *P*_*sex19*_ (default 0.2 per day) is the probability of having coitus if *AveAge* = 19.

### Age- and sex-dependent probabilities of transmission

We assumed that women have a higher (default 2-fold) per act probability of infection [11]. Following an adaptation of data from Hughes *et al*. [31] used in our previous studies [33–35], we also assumed that the relative per-act risk of transmission is higher when the susceptible partner is young (default: RR 1.492 for each decade under 46).

### Age-dependent progression rates

We followed the general strategy used in our previous studies [33–35] in which the initial placement into a CD4 category (1 = CD4>500 units, 2 = 350<CD4<500, 3 = 200<CD4<350, 4 = CD4<200, 5 = died of AIDS) and the daily probability of transitioning to a different CD4 category depend on set point viral load (SPVL) and treatment status. However, we modified the SPVL-dependent probability of progression to a higher CD4 category, in the absence of ART, to reflect the slower disease progression observed in people infected at younger ages [35] (Table 1).

**Table 1:** Effect of age on progression in the absence of therapy.

Age range (years)	Time spent in >500, 350–500, and 200–350 CD4 categories
< 30	1.23-fold more time
30–35	1.08-fold more time
35–40	0.92-fold less time
>40	0.82-fold less time

### Age-specific condom usage

Based on reports of condom usage in developing countries [15–18], we modeled decreasing per act probabilities of using a condom, *P*_*condom*_, with age as follows:

$$P_{condom}=P_{condom16}*\left(Age_{50}-16\right)/\left(Age_{50}+AveAge-2*16\right)$$

where *P*_*condom16*_ (default 0.8) is the probability that two 16-year-olds will use a condom, *Age*_*50*_ (default 35) is the age at which condom usage drops to 0.5 * *P*_*condom16*_, and *AveAge* is the average age of the partners.

### Demographic setup

HIV-negative people were assumed to enter the sexually active population in our model at age 16 and die of natural causes according to age-specific mortality tables; the arrival rate was set so that the population will increase by ~1% per year in the absence of HIV-induced mortality. We set the initial age distribution so that the age distribution will be stable in the absence of HIV-induced mortality. Because our TERGM-based network model is computationally time-consuming, we set the initial population size (*N*) to 2,000. Simulations yielding interesting or important results were then re-run with *N* = 10,000 or 20,000. Since key transmission rate parameters, such as the average number of partners per person, are independent of population size for sexually transmitted diseases [42, 43], our results should generalize to larger populations.

### Treatment cascade

We assume a certain percentage of the population (default 95%) is linkable; i.e., could become virally suppressed in response to a TasP campaign. We assumed that 100% of the linkable population is tested annually but only a subset of diagnosed people, the size of which we vary in our simulations, were linked to effective care, resulting in a suppressed viral load. In our default runs, individuals remained in care indefinitely even if they were originally linked to care due to a factor (e.g., youth) that no longer applies.

### Rollout of ART prior to targeted TasP campaigns

To establish the presence of suppressive ART in the population prior to our targeted TasP campaign, we modeled both gradual and sudden rollout schemes where the number of people receiving suppressive ART increases linearly from zero treated, starting either 9 years (gradual) or 1 year (sudden) prior to the targeted TasP campaign, to

$$S_{targ}I_{pre}/2,$$

where *S*_*targ*_ is the percentage of people who will be targeted in response to the subsequent targeted TasP campaign, *I*_*pre*_ is the number of infected people at the start of the pre-TasP rollout. The “/2” in the equation above signifies that the subsequent TasP campaign will roughly double the number of people receiving suppressive therapy in our model. Under both the gradual and sudden pre-TasP rollout schemes, random subsets of agents received suppressive ART. While gradual rollouts are more realistic, sudden rollouts allow for more precise statements about the percentage of people receiving ART at the start of the TasP campaign. That is, under a gradual rollout, the percent of people receiving ART prior to the TasP campaign is a complex function of other dynamic variables (e.g., the age distribution and the percentage of people in the two relationship-length groups). We focused, therefore, on sudden pre-TasP rollouts for summary figures with *S*_*targ*_ in the *x*-axis.

### Parameter tuning

While most of the parameters are based directly on data, we adjusted the maximum probability of infection (default 0.0025), mean degree (default 0.75), and the male- and female-specific concurrency parameters (defaults 0.25 and 0.04) to give prevalence and incidence values and male-to-female infection ratios that fall within ranges seen in sub-Saharan epidemics. [1, 2, 26, 44].

### Simulated TasP campaigns

Following the pre-TasP ART rollout, we introduced a targeted TasP campaign that increases the number of HIV+ people receiving suppressive ART ~2-fold to *S*_*targ*_*I*_*0*_, where *I*_0_ is the number of infected people at the start of the TasP campaign. (This will be an exact doubling if the number of infected people does not change between the start of the pre-TasP rollout and the start of the TasP campaign). To allow us to make more precise statements about the percentage of people treated, we stipulated for our default runs that the TasP campaign was implemented instantly (as we did for the pre-TasP rollout). After the TasP target has been hit, the *absolute number* of individuals receiving suppressive ART was assumed to grow annually at rate *r* (default 2% per year) to account for population growth and general increases in public health expenditures. That is, we capped the number of people receiving ART at *S*_*targ*_*I*_*0*_(1+*r*)^*t*^, where *t* is the number of years since the start of the TasP campaign.

Under the targeted TasP campaigns, different categories of individuals were targeted for linkage to effective care in different scenarios. Targeting strategies included: age (e.g. “under age 25”), immunological status (e.g., “CD4<500”), combinations of age and immunological status (e.g., “Under 25, CD4 < 500”), viral load (e.g., “SPVL”), and no targeting at all (“random”) (Table 2). Several of the strategies include a targeting hierarchy within the primary target range. “Under age 30”, for example, targets those under age 25 first, and then those between ages 25 and 30. In these cases, agents are linked to care at random within each successive target group until the overall treatment limit is reached. To ensure that equal numbers are treated under all strategies (prior to one strategy linking 100% of people to effective care), all strategies included a final “random” (untargeted) component that is applied once all of the people in the target groups have been linked to effective care.

**Table 2:** Key TasP strategies modeled in this paper.

Strategy	Description
Random	Select diagnosed but virally unsuppressed agents (DUs)* at random.
“CD4 < 500”	First select DUs whose CD4 count has ever fallen below 500 cells/μL.^#^ If resources are sufficient to link all selected agents to effective care, select additional agents at random until the treatment limit has been reached.
“Under age 25”	First select DUs under 25. If resources are sufficient to link all selected agents to effective care, select additional DU agents at random until the treatment limit has been reached
“Under age 30”	First select DUs under 25, then those under 30, then at random until the treatment limit has been reached.
“Under age 35”	First select DUs under 25, then those under 30, then those under 35, then at random until the treatment limit has been reached.
“SPVL”	First select DUs with SPVLs^#^ over 6.0 RNA copies/ml; then those with SPVLs less than 5.5, 5.0, 4.5, and 4.0 RNA copies/ml, respectively; then at random until the treatment limit has been reached.^&^
“CD4 < 500, Under 25”	First select DUs whose CD4 count has ever fallen below 500^#^. Once all DU agents with CD4 nadirs below 500 are linked to effective care, select agents under age 25. Once all DUs in these two groups have been linked to effective care, select additional DUs at random until the treatment limit has been reached.
“Under 25, CD4 < 500”	First select DUs under age 25, then whose CD4 count has ever fallen below 500.^#^ Once all DUs in these two groups have been linked to effective care, select additional agents at random until the treatment limit has been reached.
“Men under 30 & women under 25”	First select male DUs under 30, then female DUs under 25, then at random until the treatment limit has been reached.

* Agents just initiating suppressive therapy are classified as suppressed. Agents dropping out of therapy are assumed to remain suppressed for 30 days.

# By targeting agents based on factors that do not change during therapy, we avoid viral load and CD4 oscillations associated with having to wait for CD4 counts to drop (or viral loads to rebound) following a therapy cessation before re-initiating therapy.

& This strategy takes advantage of the fact that viral loads vary widely from person-to-person [34–35, 45]. See technical methods for details.

In the era of test-and-treat, the treatment strategies employed in Sub-Saharan African countries now most closely reflect the randomized treatment model we use in the United States. We included two additional non-random strategies, SPVL- and CD4-based targeting in order to understand how age-based strategies perform against other strategies that have the potential to outperform random (untargeted) TasP. Doing so allows us to separate the general effect of having a structured, targeted campaign from the specific effects of focusing on younger age groups. We are aware that SPVL, as defined in Table 2, has never been used and that CD4-based targeting, while once used, is no longer recommended. We are, furthermore, aware that “CD4<500” and “SPVL”, respectively, assume access to information that will be inconsistently or rarely available.

For the simulations with “sudden” pre-TasP rollouts, we quantified impacts as a function of *S*_targ_, the percent of infected people linked to effective care in response to the TasP campaign. We note that *S*_*targ*_ gives the percentage of HIV+ people receiving suppressive therapy at a single point in time (i.e., just after the TasP target has been hit). For a TasP campaign that succeeds in reducing the number of untreated people, the percentage of HIV+ people receiving ART will exceed *S*_*targ*_ as the treatment expands at the annual post-TasP rate, *r*. For a TasP campaign that fails to reduce the number of untreated people, the percentage of HIV+ people receiving ART will remain at or fall below *S*_targ_ depending on how fast the epidemic expands compared to the post-TasP expansion rate *r*.

For each value of *S*_targ_, we quantified the impact of TasP campaigns using: mean incidence 20–25 years after campaign initiation; the total number of AIDS deaths and person-years of therapy during the first 25 years of the campaign; the percentage of the population initiating suppressive ART that was *not* a part of a target group; and the percentage of untargeted infected people receiving suppressive ART. Since strategies that do not suppress viral load in a significant percentage of people outside the target group could be considered unethical, we used the two last measures as screening tools to help us decide which strategies to investigate in more detail.

For strategies for which we noted a conflict between short- and long-term AIDS death rates, we calculated time-discounted AIDS deaths—a measure that quantifies the tendency of people to prefer strategies that minimize deaths in current years over strategies that minimize deaths in later years.

We also considered a gradual version of the TasP rollout, in which the absolute number of people being treated increased linearly (without any cap) beginning 9 years before the start of the targeted TasP campaign. To prevent 100% of newly treated people belonging to a target group in the first few years of the TasP campaign, we modified these gradual simulations so that 40% of newly treated people were automatically treated at random and the other 60% were treated according to the targeting rules in Table 2 after the start of the TasP campaign. This capped the percentage of newly treated people who were treated specifically because they belonged to the target group at 60% of HIV+ people receiving *ART*.

### Sensitivity analyses

To test the dependence of our results to parameter values, we conducted a series of sensitivity analyses in which we altered or removed parameters. For these sensitivity analyses, we focused on the TasP target (*S*_targ_ value) needed to reduce long-term incidence (i.e., incidence 20–25 years after the start of the TasP campaign) 20-fold compared to a no-ART control. In preliminary work, we found that this 20-fold reduction allows for more meaningful comparisons than an absolute cutoff (e.g., incidence less than 0.1 infections/100 person-years) because parameter changes can dramatically change the baseline incidence rate. Given our 2% annual post-TasP increase in viral suppression, we found in preliminary work that a 20-fold reduction in incidence led to eventual viral eradication in all of the scenarios studied here. For sensitivity tests in which the parameter perturbation resulted in a substantially weakened epidemic, we increased an unrelated parameter (baseline transmission rate) to create a more realistic epidemic and reduce run-to-run variation.

As a part of these sensitivity analyses, we explored the effect of allowing 10% of treated individuals to discontinue treatment each year [46–49]. We did not include this in the default model because members of a target group who discontinue ART are more likely to be relinked to care than those who are not. This effect could, in principle, cycle an increasing percentage of people into the target group into ART as people outside the target group drop out.

### Statistics and replication

Error bars in the figures represent standard deviations using *n*-1 degrees of freedom where *n* is the number of replicates. Experiments shown in the figures and tables were replicated either 16 (default) or 32 (marked by ++ signs in the summary table below) times. For some of the experiments described in the text, we replicated the simulation 120 times.

## Results

### Epidemic

**Fig 2:**
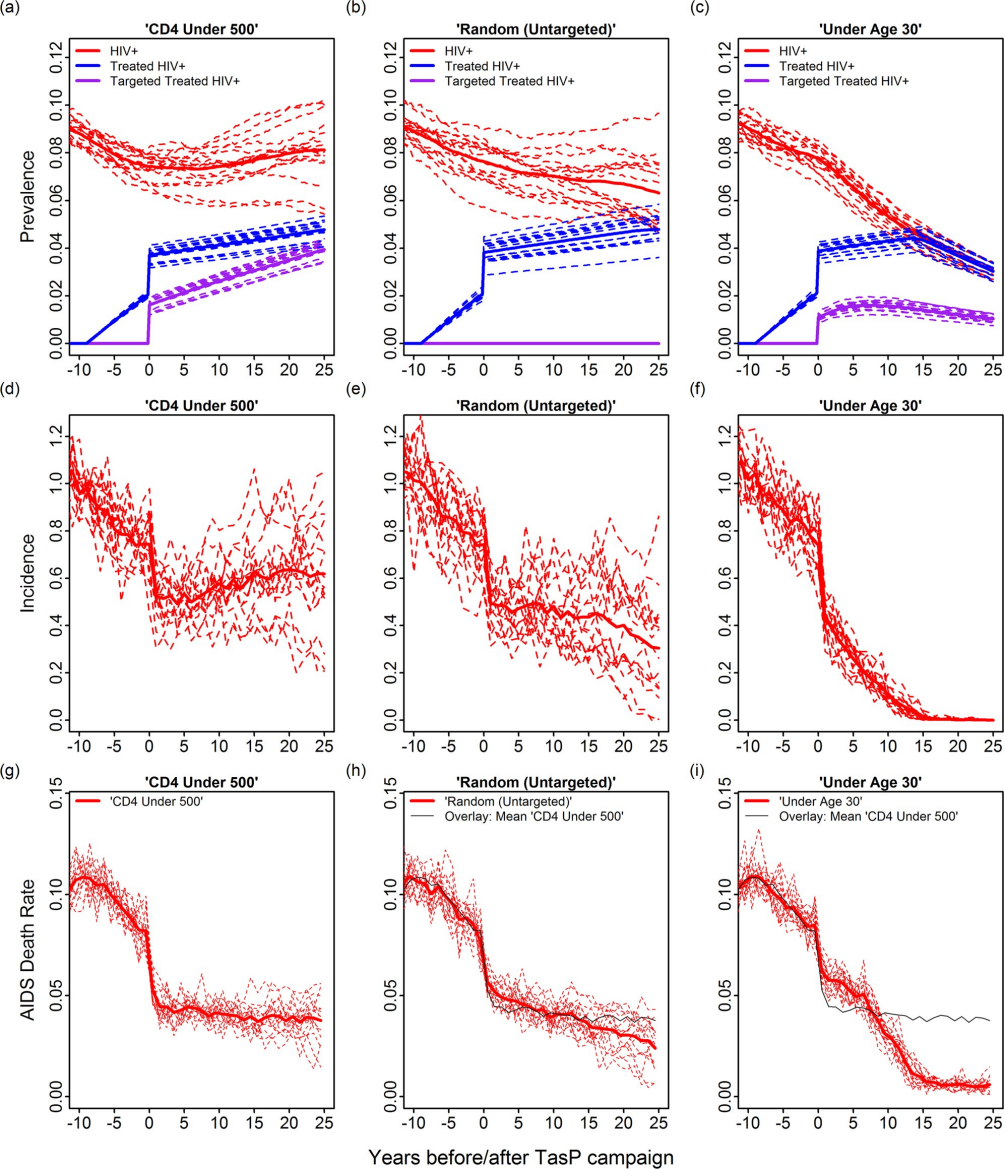
**Simulations showing the percent of the population that is HIV+ (panels a-c, red lines), HIV+ and receiving treatment (panels a-c, blue lines), and HIV+ and receiving treatment specifically due to being part of a target group (panels a-c, purple lines); incidence (panels d-f); and AIDS death rates (panels g-h) following our “CD4<500” (left-side), “random” (middle), and “under age 30” (right side) treatment-as-prevention (TasP) strategies.** To account for existing treatment, we assume a linear increase in the number of people receiving suppressive therapy beginning nine years before the TasP campaign. The TasP campaign immediately increases the percentage of HIV+ people receiving therapy to ~60%. Once the TasP campaign starts, the model uses the CD4-, random- or age-based strategies to link a subset of unsuppressed diagnosed people to care. The 2%/yr increase in the number treated after the campaign reflects population growth and generalized increases in health care efficiency or expenditures. Thick lines give the mean of 16 independent replicates; thin dashed lines show individual runs. The decline in prevalence of treated people after year 15 in panel c (blue lines) occurs because prevalence decreases once all infected agents are treated. The black lines in panels h and i give the means from the CD4<500 simulations in panel g (to highlight differences between short- versus long-term effects of “CD4<500” and “Under age 30”). For these simulations, we set the initial population size to 20,000 to reduce run-to-run variation.

Our base model gives a pre-TasP epidemic that reproduces broad features of sub-Saharan African epidemics [1, 44], namely: an initial prevalence of ~10% and a decline in incidence, though not necessarily prevalence, following a ramp-up in treatment (Fig 2A and 2B, years -0 to 10). Prior to the TasP campaign reducing incidence, the model predicts 2- to 3-fold higher prevalence in young women than young men and higher prevalence in people between 25 and 50 than in people under 25 or over 50, as reported by Shisana *et al*. [26]. In our model, women under 30 tend to get infected by older men (S1 Fig), while men over 30 tend to get infected by younger women (S2 Fig). Importantly, our model predicts that adolescents will typically get infected by partners who are under 30 (S1 Fig). This suggests that treating people under 30 could help to protect uninfected adolescents. Consistent with data in ref [50], our base model predicts a median inter-infection time of ~2.4 years (S3 Fig). Although considerably lower than it is in the presence of a very-high-risk group (see the section on sensitivity analyses below), our baseline model has a greater-than-random percentage of agents with many partners (S4 Fig).

### Advantages to youth-focused TasP

We compared age-based TasP strategies to untargeted TasP (random), a theoretical strategy (SPVL-based) and a historical (CD4-based) strategy (see Table 2 for definitions). In Fig 2 we show the number of infected, the number treated, incidence, and AIDS death rates under the “CD4<500”, “random”, and “under age 30” targeting strategies in a simulation in which ~60% of infected people received suppressive therapy as a result of the TasP campaign. In contrast to the “CD4<500” strategy and random (untargeted) TasP, where the final mean incidences were ~0.65 and ~0.3, respectively, incidence in the “under age 30” strategy dropped to less than 0.1 infections/100 person-years in all (16/16) replicate simulations ~15 years after the start of the TasP campaign.

The increase in prevalence under the "CD4<500" targeting strategy in Fig 2A is due primarily to HIV-infected people living longer and the failure of this strategy to reduce incidence to zero. However, we note that demographic shifts (AIDS deaths reducing the number of people between ages 30 and 50) also contribute to increasing prevalence in this and other simulations. This attrition increases the proportion of people who have a higher risk of getting infected (i.e., young adults) in the first 20 years of the TasP campaign. Also, because our baseline model includes comparatively favorable conditions for the spread of virus, the average SPVL increases by about ~0.1 logs between years 0 and 20 (due to a well-known evolutionary tradeoff verified in earlier versions of our software [34]). This small increase in the average SPVL contributes somewhat to the increasing prevalence during this period.

The purple lines in Fig 2A and 2C give the percent of the population that was treated specifically due to their having CD4 counts below 500 and being under age 30, respectively. For the “Under age 30” strategy, the percent of treated people who were treated specifically due to being <30 peaked at ~38% after ~6 years, then declined slowly to ~32% at year 15 (after which ~100% of people were virally suppressed). For the “CD4<500” strategy, the percent of treated people who were treated specifically due to having a low CD4 count increased monotonically from ~44% at year 1 to ~83% at year 25. This indicates that the “under age 30” strategy succeeded in reducing incidence despite being significantly less exclusionary that the historical “CD4<500” strategy.

**Fig 3:**
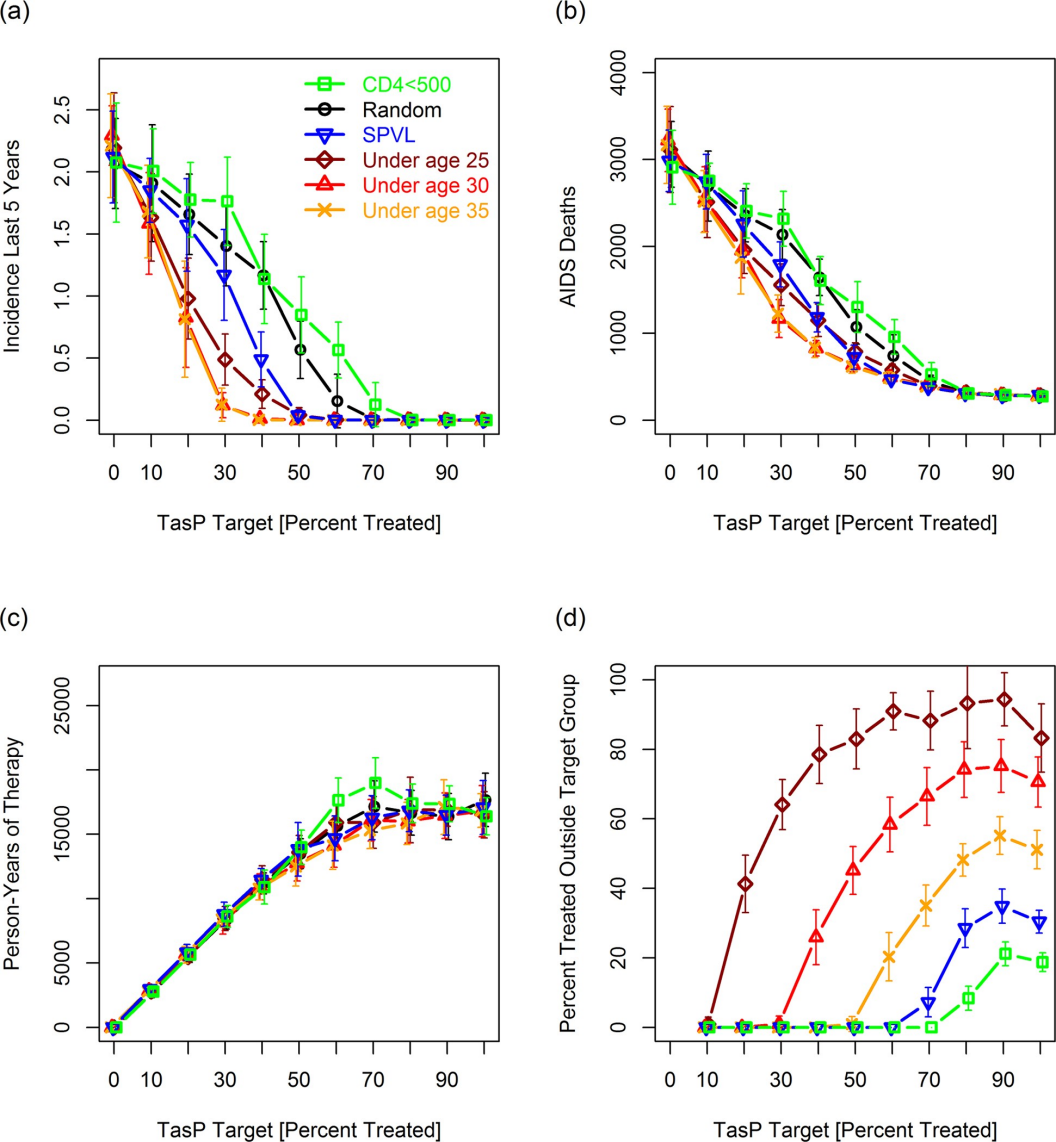
**Effect of targeting strategy and the TasP target (*S***_***targ***_**) on: (a) incidence 20–25 years after the TasP campaign, (b) AIDS deaths between years 0 and 25, (c) person-years of therapy (included to demonstrate that the age-based strategies did not inadvertently result in more people being treated), and (d) the percentage of HIV+ people initiating treatment at the start of the TasP campaign who were not a member of a target group.** For random (untargeted) TasP, the values in panel d will always be 100% (data omitted from graph). The apparent decline in panel d between 90% and 100%, a decline not seen in other experiments, reflects statistical noise accentuated by the fact that only 95% of the population is linkable in this simulation (i.e., *S*_targ_ = 100% translating to 95% suppression). Each point is the mean of 16 replicates. Bars give standard deviations (*SD*s). For normally distributed data, 95% confidence intervals would be ~55% the width [since *t*_0.025,15_ *SD/sqrt(15) = ~0.55*SD]. For this simulation, we set the initial population size to 10,000. In this and subsequent figures we assumed sudden pre-TasP rollouts so that the TasP campaign will roughly double the number of virally suppressed people.

Fig 3 presents results for a broader range of strategies and for multiple values of *S*_targ_ (percent infected people linked to effective care in response to the TasP campaign) demonstrating the range of scenarios over which age-based strategies convey an advantage. Fig 3A (average incidence 20–25 years after implementing the TasP campaign) shows that *S*_*targ*_ had to be set to 70% and 80%, respectively, to reduce incidence rates to negligible levels for the “random” and “CD4<500” targeting strategies. Under the age-based targeting strategies, by contrast, incidence could be reduced to negligible levels using *S*_*targ*_ values of 40% to 60%. Age-based strategies also resulted in fewer AIDS deaths over 25 years than random or “CD4<500” (Fig 3B). The SPVL strategy gave results comparable to the age-based strategies for *S*_targ_ > = 50%, but somewhat worse results for *S*_targ_ < 50%. With the exception of the “CD4<500” strategy, which resulted in more person-years of therapy for *S*_targ_ between 60 and 80%, the total number of person-years of treatment over the course of the simulation was similar for all strategies (Fig 3C). This shows that the success of the age-based strategies is not due to these strategies treating more people. In particular, it shows that this success is not due to the longer lifespan of younger people starting therapy (relative to old people starting therapy) increasing the number of person years of therapy.

Fig 3A and 3B also show that the benefit of age-based targeting depends on the percentage of the overall population that can be virally suppressed. For high levels of suppression (*i*.*e*., *S*_*targ*_ > = 80%), the advantage to age-based targeting largely disappears. A similar effect can be seen using our alternative (gradual) rollout strategy in which the number of people being treated increases linearly with time: age-based targeting is highly effective when rollouts are slow or moderate (S5 Fig, left and middle columns); however, the advantage to age-based targeting disappears when treatment ramps up quickly (S5 Fig, right column).

We note that Figs 2 and 3 assume that 95% of any given target group could be reached during the treatment campaign. We kept this value high in order to demonstrate the underlying potential of age-based TasP. In the Sensitivity Analysis section below, we report on what happens when this percentage is altered.

### Inclusivity / exclusivity of strategies

A targeting strategy that focuses the majority of resources on a single group is not an acceptable strategy due to the ethical issues of potentially leaving a proportion of the population without access to care. Of the five “non-random” strategies in Fig 3, the percentage of the HIV+ population initiating suppressive therapy at the start of the TasP campaign that was *not* a member of a target group was highest under the “under age 25” and “under age 30” strategies (Fig 3D).

For *S*_*targ*_ = 60% and the “under age 25” strategy, for example, ~87% of people initiating suppressive therapy at the start of TasP campaign fell outside the target group—a result that can be attributed to there being relatively few HIV+ people under the age of 25. For the “under age 30”, “under age 35”, “SPVL”, and “CD4<500” strategies, the percentages outside the targeted group were 58%, 35%, 7% and 0%, respectively (Fig 3D). If we were to include people who were virally suppressed prior to the TasP campaign, the percentage of treated people who were not a member of the target group for the “under age 25”, “under age 30” “under age 35”, “SPVL”, and “CD4<500” strategies, would jump slightly (to ~90%, ~70%, ~50%, ~25%, and ~10%, respectively). In subsequent simulations, we focus mainly on comparing the “under age 25” and “under age 30” strategies and variants thereof with the “random” and/or “CD4<500” strategies.

The *y*-axis in Fig 3D is similar to *S*_*tar*g_ in that it quantifies “inclusivity” at a single point in time (i.e., just after the TasP target has been hit). For TasP campaigns that reduced prevalence, the percentage of *untargeted infected individuals receiving suppressive therapy* increased over time (as can be inferred from the narrowing difference between the red and blue lines in Fig 2C). However, for the TasP campaigns that failed to reduce prevalence, the percentage of untargeted individuals receiving suppressive therapy either decreased or remained roughly constant (Fig 2C before prevalence flattened around year 20) or in subsequent years. In other words, the successful TasP campaigns became more inclusionary over time, while unsuccessful TasP campaigns either became more exclusionary or retained similar levels of inclusivity over time.

Fortunately, our simulations suggest that one will not have to wait long to find out whether a campaign will succeed. For the successful “under age 30” strategy shown in Fig 2, for example, we observed a ~2-fold drop in incidence within 2 years of starting the TasP campaign. While drops in incidence are unlikely to be as rapid under more realistic scale-ups, incidence is a highly sensitive measure that can, with the aid of an epidemiological model, predict whether an in-progress TasP campaign is likely to reduce prevalence. In cases where incidence cannot easily be calculated, age-based TasP can be assessed from HIV prevalence in cohorts of young people who become sexually active after starting the TasP campaign. For our “under age 30” strategy in Fig 2, for example, we observed a 4.8-fold drop in prevalence in those under 25 six years into the TasP campaign.

Increases in *S*_targ_ above a specific threshold (given by points in Fig 3D in which the lines intersect the *x*-axis) result in treatment being directed almost entirely to people outside the target group. For the “under age 25” and “under age 30” strategies we observed continued benefits (e.g., reductions in AIDS deaths) as *S*_targ_ increased beyond this threshold (Fig 3A and 3B). For the “under age 25” strategy in Fig 3A, for example, we observed lower incidence and fewer AIDS deaths for *S*_targ_ = 20% than for *S*_targ_ = 10% (the value of *S*_targ_ at which all reachable agents under the age of 25 have been linked to care). While not surprising, this is important for broader discussions about age-based TasP (see last paragraph of discussion).

### Time-discounted AIDS death rates

While age-based targeting greatly reduces the number of deaths over the long-term, it comes at the cost of slightly more AIDS deaths relative to CD4-based targeting in the years immediately following the TasP campaign (as illustrated in Fig 2I). This tradeoff can be quantified using time-discounted AIDS deaths; i.e., a measure of AIDS deaths that gives less weight to future deaths than current deaths. In S6 Fig, we show that, for discount rates between 0 and 7% per year, age-based targeting reduces time-discounted AIDS deaths compared to the other strategies in Fig 3.

### Sensitivity analyses

**Fig 4:**
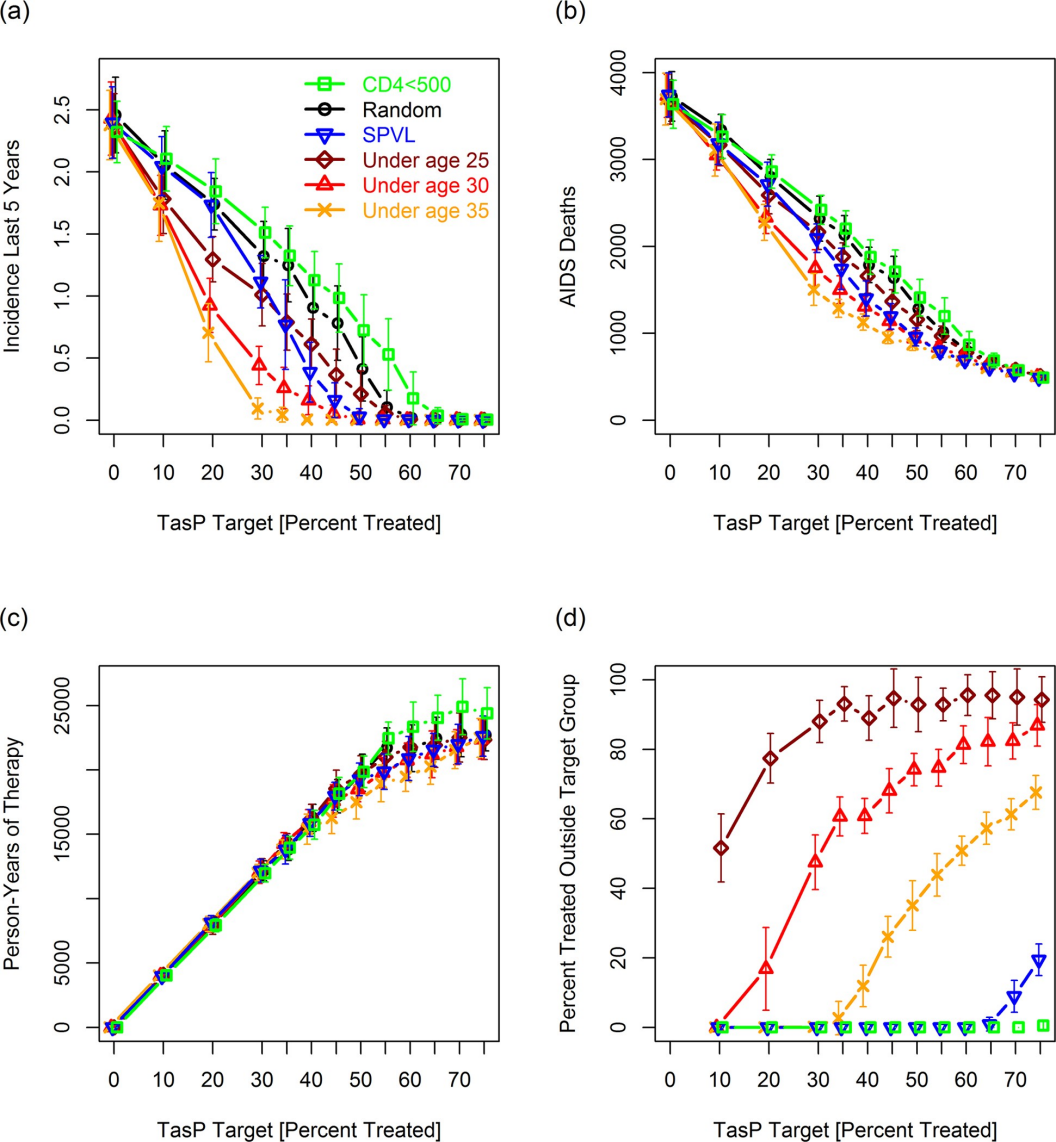
Performance of age-based TasP in sensitivity experiment in simulations [Table 3, perturbation 14] in which *all* of the primary age-related risk factors except for age-related homophily were removed (i.e., in a model in which relationship durations, transmission rates, and probabilities were all independent of age). (a) Incidence 20–25 years after the TasP campaign. (b) AIDS deaths between years 0 and 25. (c) Person-years of therapy. (d) Percentage of HIV+ people initiating treatment at the start of the TasP campaign who were not a member of a target group. Error bars present standard deviations based on 32 replicates.

To test the extent to which our results depend on parameter values and functional forms, we conducted a series of sensitivity analyses in which we altered parameters identified *a priori* as being likely to contribute to the success of age-based TasP targeting (see methods for details). In Table 3, we demonstrate continued benefits of age-based targeting when: (i) the probability of transmission was increased; (ii) all agents have the same average relationship duration; (iii) young and old people have the same coital frequency; (iv) young people no longer have a higher per-act risk of infection; (v) the average age difference between male and female partners increased to 8 years; (vi) the average difference in ages after accounting for the male-female age difference has increased to 8 years; (vii) the age-related homophily term was removed in its entirety so that young people no longer preferentially form relationships with other young people; (viii) the percentage of people who could potentially be linked to HIV-1 treatment services was decreased; (viii) testing rates were varied; (ix), treated people have a small per-day probability of discontinuing treatment; (x) all agents have the same SPVL, and (xi) in a triple-perturbation experiment in which relationship durations, transmission rates, and probabilities of coitus were all independent of age (Fig 4 and Table 3, perturbation 14) (though in this case only "Under age 30" and "Under age 35" performed substantially better than untargeted TasP). We also observed benefits to age-based TasP when condom use was independent of age, though this is not surprising since our baseline model assumes that young people use condoms more often.

**Table 3:** Sensitivity experiments in which we varied key age-related and epidemiological parameters.

Parameter type	Perturbation	Final Incidence (no tx)*	*S*_targ_ to reduce incidence 20-fold^%^ (Untargeted)	*S*_targ_ needed to reduce incidence 20-fold (Percent relative to untargeted TasP^&^)				
				CD4 <500	High SPVL	Under age 25	Under age 30	Under age 35
Baseline	1. Baseline (*N* = 10,000^++^)^||^	1.9	60	117	83	75	50	50
Relationship Durations	2. All agents have the same relationship duration (*D*_1_ = *D*_2_ = 1095, *N* = 10,000^++^)	3.7	65	115	77	77	62	62
	3. Group 1 agents at very high risk and do not transition to group 2. Group 2 has a slightly higher risk. (*D*_1_ = 10, *D*_2_ = 2190, *N* = 10,000^++^)	3.1	60	108	83	75	58	50
	4. Group 1 larger and at high risk (42% enter into group 1, *D*_1_ = 100, *D*_2_ = 3650, *N* = 10,000^++^)	17	85	100	88	82	82	82
	5. Group 1 larger and at very high risk (42% enter into group 1, *D*_1_ = 10, *D*_2_ = 3650, *N* = 10,000^++^)	28	90	100	89	89	89	89
	6. Group 1 is somewhat larger and does not transition to group 2, both groups 1 and 2 have higher risks. (25% enter into group 1, *D*_1_ = 100, *D*_2_ = 365, *N* = 10,000^++^)	30	--	--	--	--	--	--
Prob Sex	7. *P*_sex_ independent of age (*P*_sex_ = 0.14, *N* = 10,000)	1.3	55	114	82	82	55	55
	8. *P*_sex_ independent of age (*P*_sex_ = 0.20, *N* = 10,000)	3.4	70	107	78	78	57	50
Transmission rate	9. Higher transmission rate (Trans 2x^#^, *N* = 10,000^++^)	8.2	85	100	82	76	65	59
	10. Transmission rate independent of age (Trans 2x^#^, *N* = 10,000^++^)	6	75	106	80	86	66	53
Age-related homophily terms	11. Male-female age difference doubled to 8 years (*N* = 10,000^++^)	2.0	65	108	77	77	46	46
	12. Average age difference doubled ^@^ (Trans 1.33x^#^, *N* = 10,000)	2.9	65	108	92	76	69	61
	13. Age-related homophily removed (Trans 2.5x^#^, *N* = 10,000^++^)	2.8	55	109	100	91	82	73
Removal of all youth-related risk factors	14. Durations (*D*_1_ = *D*_2_ = 1095), *P*_sex_ (0.2) and transmission rate (Trans 1.33x) all independent of age. (*N* = 10,000^++^)	2.4	65	109	90	100	82	55
	15. Repeat of 14, with no age-related homophily (*N* = 10,000^++^)	2.0	45	111	100	102^$^	100	100
Percentage that could be linked to care	16. Maximum set at 80% (*N* = 10,000^++^)	2.1	60	108	83	83	66	50
	17. Maximum set at 70% (Trans 1.33x^#^, *N* = 10,000^++^)	4.4	65	108	100	100	85	77
	18. Maximum set at 60% (*N* = 10,000^++^)	2.2	60	100	91	91	75	67
Condom Usage	19. Condom usage independent of age	2.7	70	114	86	71	57	57
SPVL Variation	20. Variation in SPVL set to zero (*N* = 10,000)	3.1	65	108	100	77	54	54
Testing rate	21. Testing only every 3 years (*N* = 10,000^++^)	2.0	70	100	86	86	64	57
Discontinuation	22. Agents drop out (*P*_drop_ = 10%, Trans 33% higher^#^)	3.7	80	100	75	75	50	50
			Color legend: Performance of targeted strategy relative to untargeted					
			Better	Same				Worse
			< 65	65–80	80–95	95–105	105–120	>120

* Incidence 20–25 years after the start of the TasP campaign in which no one was treated. Both this value and the values for *S*_targ_ needed to reduce incidence 20-fold will vary from experiment to experiment (i.e., from row to row) due to chance fluctuations in the starting networks.

^++^ Experiments with 32 replicates, those without this marker had 16 replicates.

% Having an average incidence between years 20–25 that is at least 20-fold lower than in it would be in the absence of ART. Values in the column are rounded to the nearest 5%. The numbers in this column and the columns to the right are all percents.

& Values equal 100* *S*_*targ_targeted*_*/S*_*targ_untargeted*_. The 50% in top-right box of the table, for example, indicates that long-term incidence can be reduced to low levels with an *S*_targ_ value of 30 with the “Under age 35” strategy (since 50% of 60 is 30). Most of these values are accurate to around 5%.

|| This is a "high *N*" replicate of the experiment in Fig 3 that includes additional values for *S*_targ_ between 30 and 90, but fewer between 0 and 30.

-- Incidence failed to drop 20-fold even with *S*_targ_ = 100%

# Baseline transmission rate increased in order to reduce run-to-run variation.

@ Average difference between partner ages *after* accounting for the male-female age difference.

$ Additional *S*_targ_ values were examined to more precisely determine percent differences from random untargeted TasP.

To keep our focus on normal age-related risks, we did not include a very-high-risk group in our base model. To explore the effect of adding a "very-high-risk" group to our model, we did a series of perturbation experiments in which we manipulated relationship durations for groups 1 and 2, made these groups age-independent, and/or changed the proportion of agents in the two groups. Table 3 (perturbations 3–6) gives some representative results. We found that age-based TasP greatly outperformed random (untargeted) TasP when members of group 1 have very high risks and do not transition to group 2 (Table 3, perturbation 3), as well as when a greater percentage of people belong to group 1 and in which a group 1 has moderately or greatly elevated risks (Table 3, perturbations 4 and 5, respectively). (We note that the coloring in Table 1 understates effects when percentages are high: in perturbations 4 and 5 age-based TasP doubled the number of people who did *not* have to treated in order to drive incidence to zero.) These perturbations, therefore, show that age-based TasP can be highly effective in a model with a risk group that results in a heavy-tailed partnership-distribution curve (S4 Fig).

However, when we pushed incidence even higher by reducing relationship durations in both groups and increasing the proportion of agents in group 1 (Table 3, perturbation 6), we found that none of the strategies could reduce incidence to zero despite 100% of eligible agents (i.e., 95% of all agents) receiving suppressive therapy. While incidence was unrealistically high, this establishes that TasP (age-based or not) is not assured of controlling the epidemic.

Age-based TasP also failed to provide an advantage over untargeted TasP in a “zero age risks” perturbation in which we eliminated age-related homophily and made relationship durations, transmission rates, and probabilities of coitus independent of age (Table 3, perturbation 15). While not surprising, this provides re-assurance that age-based TasP did not outperform the other strategies due to some unappreciated aspect of our model.

Contrary to our initial expectations, SPVL variation had little effect on the efficacy of age-based TasP (Table 3, perturbation 20). We found that untreated HIV+ people under the age of 30 have SPVLs that are roughly double those of untreated HIV+ people over 50, an increase that should translate to ~25% increase in the infectivity of untreated young people. However, this is proved to be fairly small compared to other age-based risks shown to have modest effects in Table 3. The probability of sex, for example, will be roughly halved by age 50, and yet we continued to a large advantage to age-based TasP when the term for age-based coital frequency was removed from the model (Table 3, perturbations 7 and 8).

**Fig 5:**
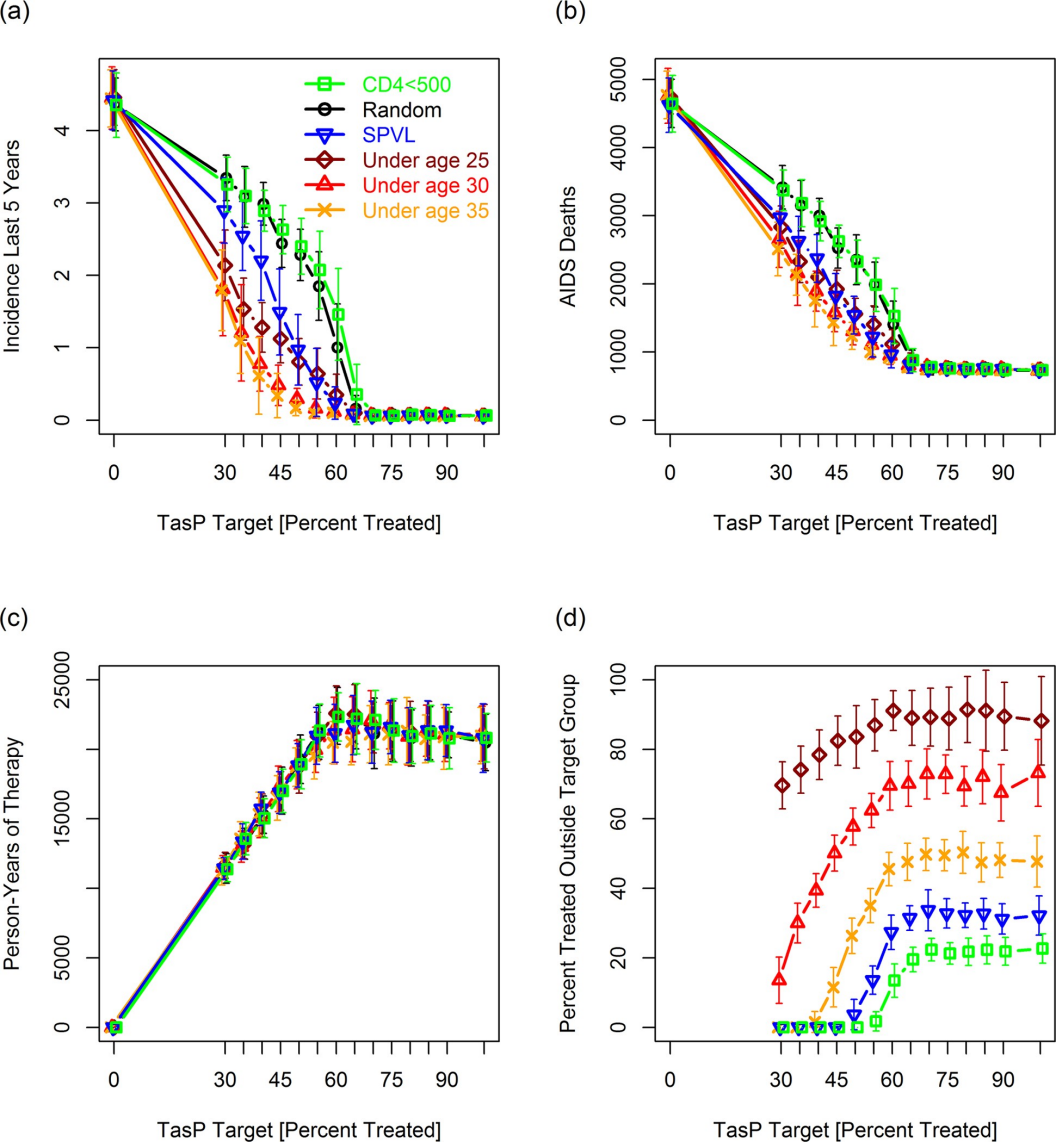
Performance of age-based TasP in simulations [Table 3, perturbation 17] in which only 70% of HIV+ people could be linked to care (i.e., only 70% would get tested and treated under a vigorous treatment campaign). (a) Incidence 20–25 years after the TasP campaign. (b) AIDS deaths between years 0 and 25. (c) Person-years of therapy. (d) Percentage of HIV+ people initiating treatment at the start of the TasP campaign who were not a member of a target group. Error bars present standard deviations based on 32 replicates.

The largest and most informative reductions in the efficacy of age-based TasP relative to random (untargeted) TasP occurred in perturbations in which we removed the age-related homophily term (Table 3, perturbations 13 and 15) and in which we reduced the maximum percentage of people who could be linked to care to 60–70% (Table 3, perturbations 17 and 18 and Fig 5). We note that neither age-based homophily nor overall linkage to care directly affects the susceptibility of a young person. Instead, they affect the probability that a young person’s *partners* will be infectious. Together these results point to a substantial contribution of age-specific herd immunity (ASHI) to the success of age-based TasP: in order for ASHI to work, a sufficient percentage of the partners of HIV- adolescents entering the sexually active population need to be virally suppressed.

While decreasing the maximum percentage of people who could be linked to care reduced the efficacy of age-based TasP, we found it remarkable that age-based TasP could succeed (albeit not as dramatically) in a model in which only 60% to 70% of agents could be linked to care (Fig 5 and Table 3, perturbations 17 and 18). Three factors that contributed to this success. First, the percentage of people who are not linkable decreases over time in the face of HIV-induced mortality. Second, and more interestingly, surviving HIV+ people who are not linkable became less infectious over time due to increases in their average age (since age-based TasP reduces incidence in young people) and reductions in their average viral load (since people infected with high SPVL viruses die sooner in the absence of therapy).

In most of the simulations in Table 3 where the value of *S*_targ_ needed to drive incidence to zero following age-based TasP was the same or only slightly lower than random (untargeted) TasP, we continued to see advantages to age-based TasP for lower values *S*_targ_. For *S*_targ_ < 55 in Fig 5, final incidence and total AIDS deaths continued to be lower for the age-based strategies than for our “random” and “CD4<500” strategies. For some of these simulations, “SPVL” (which doesn’t depend on ASHI) reduced final incidence and AIDS deaths more than "under age 25” for selected *S*_targ_ values. However, we have not considered “SPVL” further because it is, as defined in Table 2, a theoretical strategy introduced for purposes of comparison. “SPVL” furthermore did not perform as well as “under age 30” in our baseline simulations (Fig 3A and 3B), and is considerably more restrictive (Figs 3D, 4D, and 5D).

### Hybrid strategies combining age with CD4 status or sex

Our analyses have focused so far on strategies that consider age, sex, and CD4 status in isolation. We also studied strategies that integrated these in various combinations. Although we hypothesized that combining age and CD4 status for targeting might yield even better results than either factor in isolation, we did not see any clear advantages to strategies combining “under age 25” and either “CD4<500” or “CD4<200.” (S7 and S8 Figs). [For these simulations we first considered combinations of “Under age 25” and “CD4<500”, but these strategies were so exclusive (S7 Fig) that we switched to “CD4<200”-age hybrids.] CD4-based strategies that utilized age as a secondary criterion performed about the same as those that used only CD4 status, while age-based strategies that utilized CD4 status as a secondary strategy performed about as well as strategies that used only age (S7 and S8 Figs). Although there was a weak trend for “Under 25, CD4<200” to reduce time-discounted AIDS deaths relative to “Under age 25” for *S*_targ_ between 50 and 60% using a 7% discount rate, CD4-age hybrids did not reduce time-discounted AIDS deaths using the standard 3% discount rate (S9 Fig). These tests, in other words, failed to support our hypothesis that combining age and CD4 status would yield significantly better outcomes compared to straight age-based or CD4-based targeting.

Since young women tend to get infected by older men, we hypothesized that including a higher target age for men (e.g., men < 30, women < 25) might enhance the effectiveness of age-based TasP relative to sex-independent cutoffs; however, we did not see any significant advantages of “men <30, women<25” compared to “men <25, women < 30 (S10 Fig). The “men <30, women<25” and “men <25, women < 30” strategies, while considerably better than random, generated incidence rates and AIDS deaths that fell between the “under age 25” or “under age 30” strategies.

## Discussion

Within a heterosexual epidemic with ordinary risk behaviors, our model shows that age-based TasP can halve the percentage of people that need to be virally suppressed in order to reduce HIV incidence to negligible levels. Benefits to youth-focused TasP were obtained in scenarios in which each of the age-related risk factors (e.g. shorter relationship durations in young people) were removed, in scenarios where the male-female age difference was doubled, and in scenarios in which only ~60% of people could be linked to care given a sufficiently vigorous TasP campaign. These benefits were not due to age-based TasP treating more people over time. In fact, for TasP campaigns treating more than ~50% of infected people, age-based TasP typically reduced the total number of person-years of therapy relative to untargeted TasP (Figs 3B, 4B and 5B). Sensitivity analyses revealed age-related homophily to be the biggest single driver of the success of youth-focused TasP. Age-related homophily is important because, in its presence, treatment of young people provides age-specific herd immunity (ASHI) that protects adolescents entering the sexually active population (a concept referred to as “ring immunity” by Bershteyn *et al*. [22]). Protection of these adolescents will, in turn, translate to protection for subsequent cohorts of adolescents entering the sexually active population. Over time, ASHI gives rise to an ever-expanding “AIDS-free generation” that drives HIV to extinction.

Our model does not include MSM, PWID, people with very high mean degrees (e.g. sex workers), entry of HIV+ people from other regions, and people infected with drug resistant viruses. We decided against including these groups for two reasons. First, for the sub-Saharan countries with the highest HIV-1 prevalence, HIV is predominately spread via heterosexual contact (thus reducing the need to include MSM and PWID). Second, we wanted to establish the effects of youth-focused TasP in a population with a minimum number of non-age-specific complications. Had we created a comprehensive model that included all of these groups, we would likely have ended with a complex multi-part recommendation that could have obscured the significant and potentially unique role that untreated young people play in sustaining transmission within heterosexual populations with risk characteristics of the general population. That said, we did perform supplemental sensitivity analyses showing that age-based TasP can significantly outperform untargeted TasP in the presence of both an age-independent high-risk group (Table 3, perturbation 3) and an age-dependent high-risk group (Table 3, perturbations 4 and 5). While these perturbation analyses were limited, they show that the model is not overtly sensitive to the presence of high-risk groups. The model also includes sex-specific concurrency terms that cause a higher proportion of men to have concurrent partners than women. In combination with our term for age-disparate male-female relationships, this parameter increases the proportion of men having concurrent relationships with younger women.

We note that predicted inter-infection times, furthermore, tend to be shorter (mean < 22 months) in MSM-dominated cohorts [51] than in our heterosexual model (mean ~2.4 years). Indeed, Yousef et al. [52] estimated an inter-infection time of ~6 months in an MSM-dominated transmission cluster; i.e., an inter-infection time that is considerably shorter than the typical time from infection to diagnosis. In populations like this, it is conceivable that no amount of TasP (regardless of the strategy) will be enough control the epidemic. In such cases, TasP would need to be combined with other strategies (e.g., PrEP).

Within the context of our general population heterosexual model and our goal of reducing incidence (but not prevalence) to zero, youth-focused TasP yielded the largest benefits in simulations with treatment coverage under ~70% (i.e., at levels below those that would be attained under UNAIDS 90-90-90 goals for 2020). At higher levels, incidence could be driven to negligible levels under all strategies considered here. However, there are reasons to expect that youth-focused TasP could be beneficial even in regions that have met or are on target to meet 90-90-90 goals. For populations in which virus spreads rapidly, the 73% viral suppression conferred by 90-90-90 may not be enough to drive incidence to zero. In fact, our simulations show that in the presence of a subpopulation of people with very short relationship durations that no amount of youth- or adult-focused treatment could end the epidemic despite annual testing (Table 3, perturbation 6). Even in populations in which 90-90-90 is sufficient, it may be hard to sustain treatment services for the full 50–60 years needed to eradicate the virus from the population (i.e., to get to the point where there are no sexually active infected people left in the population). At any time during this period, *R*_0_ could increase if people perceive less risk due to the declining epidemic and “let their guard down” with respect to preventative measures like condom and PrEP use. These and other considerations (e.g., uncertainties in funding; lack of an effective vaccine) dictate that public health officials should consider additional efficacies that modelers identify and demonstrate as robust in the context of broader ethical and logistical considerations identified by other stakeholders.

We had expected that setting a higher age target for men might benefit the population by protecting young women partnered with older men. However, our “men under 30, women under 25” strategy was not measurably better at reducing long term incidence than our “under age 25” and “under age 30” strategies, or one that included a higher target for women (“men under 25, women under 30"). Sex-stratified age targeting conferred few long-term benefits because treatment of young people extends to older people over time. A strategy that treated all infected women under 25, for example, would, in the absence of drop outs, cover a significant percentage of women under 30 after 5 years. While we leave open the possibility that more extensive investigations with a more detailed model will show a benefit to sex-specific age targeting, we expect any such benefits to be small compared to the large benefits that accrue to straightforward age-based targeting.

Given the inverse correlation between HIV viral load and CD4 counts we had also expected that previously used CD4-based targeting would reduce incidence compared to random (untargeted) TasP. Our simulations, however, showed, if anything, slightly worse long-term outcomes under CD4-based targeting. This can be explained, in part, by delays between infection and CD4 decline: people with low CD4 counts will, on average, be older than people with high CD4 counts. Another factor is that our model accounts for reductions in sexual activity in late-stage AIDS. CD4-based targeting, therefore, channels a greater percentage of "resources" to people who would otherwise be non-infectious due to illness. However, we severely doubt that treatment of end-stage AIDS patients (i.e., patients who are most likely to seek out care) would negatively affect the percentage of non-AIDS patients who receive therapy in real-life. The slightly worse performance of the CD4-based strategies, therefore, is, in part, an artifact of the way we set up the comparisons in our model.

Our model suggests a significantly stronger advantage to youth-focused TasP than what one might infer from previous modeling studies by Alsallaq *et al*. [21] and Bershteyn *et al*. [22]. This is due to several factors. First, we included age-related risk factors (declines in coital frequency and per-act transmission rates with age) that were not included in these previous models. It may also be explained, in part, by differences in the groups being modeled. Alsallaq *et al*. modeled the effects of prioritizing people under the age of 25. In the absence of other age-related risks, our "under age 30" and "under age 35" strategies outperform our "under age 25" strategy (Table 3, perturbation 14). We speculate Alsallaq *et al*. would have seen larger benefits had they extended their target age to 30. Bershteyn *et al*. focused on a strategy that targets those between ages 20 and 30. Although this strategy avoids the costs of testing a group with low prevalence (i.e., adolescents), it does not protect adolescents from getting infected by other adolescents. While the “ring of protection” imposed by their “ages 20 to 30” strategy is very likely to reduce the number of adolescents who get infected in the first place, the age-based strategies considered here are more robust to the risk of HIV spreading between adolescents. In addition, Bershteyn *et al*. compared their “ages 20–30” strategy to a cohort-based strategy in which a defined cohort (i.e., people entering the sexually active population during the first 10 years of the treatment campaign) was prioritized for treatment for the rest of their lives to the exclusion of others. While useful for determining what kinds of age-based strategies are likely to be the most effective, this cohort-based comparator is not so helpful for our purposes because it enhances treatment of young people during the critical early years of the treatment campaign (though, admittedly, not during the later years).

Third, we included more optimistic treatment scenarios. Bershteyn *et al*. and Alsallaq *et al*. proposed their models when public health officials had just introduced the 90-90-90 concept. In the time that has elapsed since their papers, public health officials have since introduced the 95-95-95 concept, a level of suppression what would be well within the ~80% linkage to care that optimizes age-based TasP in our model. In other words, we have taken an aspirational approach that asks what it would take to end the epidemic rather than accept the *status quo*. Finally, although the effect proved to be rather small (Table 3, perturbation 20) our model accounts for the potential for youth-focused TasP to dampen spread by selecting for less virulent viruses (since young people are less likely to have died from high SPVL viruses prior to the TasP campaign).

Because of the way we set up our comparisons, we believe that our model provides a clearer illustration of the potential of youth-focused treatment and prevention services than previous studies. Our model is also backed by extensive sensitivity analyses designed to identify the key factors responsible for the success of age-based TasP. Our model, furthermore, takes advantages of an established and well-reviewed set of social network routines that preserve key features of the network over time. Although somewhat tangential to our main hypothesis, the ability to preserve network features contributes interesting and potentially explanatory details; for example, we noted that the increase in prevalence in Fig 2A could be tied, in part, to shifts in the age distribution induced by HIV-related mortality. This shift is an example of a kind of network factor that the underlying *statnet* routines are well-equipped to handle. Bershteyn *et al*.*’*s and Alsallaq *et al*.*’s* models, however, have other strengths. Bershteyn *et al*., for example, include a greater variety of relationships, while Alsallaq *et al*. include cost and quality life-year calculations that we have not incorporated in our model. Although we differ in the magnitude of the benefit, the fact that independently derived models that include different levels of detail and methods of analysis all show advantages to age-based HIV treatment and prevention services lends support to our findings.

There are, of course, many additional details concerning age-related infection risks that could potentially impact the effectiveness of age-based treatment. Brewis and Meyer [30], for example, suggest the overall reduction in coital frequency with age is a result of strong reductions in coital frequency with age in males masking an underlying tendency for coital frequency to peak around age 29 in females. A more detailed coital frequency function could, in principle, capture this and other complexities (e.g., the effect of partnership duration on the probability of coitus) described in the sexual behavior literature. Similar complexities exist with parameters for age-dependent relationship durations, and per-act probabilities of transmissions. Our finding, however, that youth-focused TasP continues to provide large benefits in variants of the model in which key age-related parameters were completely removed indicates robustness of the model to these potential complications.

While we used time-dependent treatment limits (i.e., we assumed a “zero-sum” game) to ensure a fair comparison of strategies in the simulations, this should in no way be taken to argue for redistributing HIV/AIDS resources from “old” to “young” PLHIV (or from AIDS patients to non-AIDS patients) in order to “more efficiently” end the epidemic. We argue instead for expanded funding and efforts to increase care and treatment for all ages and CD4 counts. Our model, of course, comes at a time when testing and treatment rates in youth significantly lag behind those in older people [19]. Giving extra attention to youth-centered testing and treatment services could therefore be an equitable means of bringing the epidemic to an end in the context of an overall expansion of care. Attention to HIV infection in youth may take on a particular urgency in low- and middle- income countries where a looming demographic 'youth bulge’ [53] threatens to further strain treatment and prevention programs.

## Supporting information

S1 Text Supplementary methods. Gives an overview of the ***Evonet_HIV*** package.
(DOCX)

S1 Fig Ages of infectors of women and men under the age of 35. Each histogram shows the ages of the infectors (i.e., the ages of people who infected someone in the group shown in each histogram) in the absence of treatment. The bars show all infection events between years 0 and 5 involving infectors and recipients who were in the relevant age range at the time of infection. Each bar gives the average of 16 replicates. Red bars indicate infectors who were in the same age range as the recipients.
(TIF)

S2 Fig Ages of infectors of women and men over the age of 35. This is a continuation of S1 Fig. Axes, colors, and conditions are described in S1 Fig.
(TIF)

S3 Fig Inter-infection times. This graph shows the time elapsed between the time of infection and the time that their infector got infected during the last 25 years of a 45-year simulation without treatment.
(TIF)

S4 Fig Cumulative distribution of partnership numbers for our baseline model and selected perturbation experiments from Table 3. The *x*-axes give natural logarithm of the total number of partners per person for people. The *y*-axes give the natural logarithms of cumulative distribution starting with the agent with the most partners. To reduce variation caused by young people not having as much time to form relationships as older people, we plotted distributions for people within narrow age ranges. The red circles and blue squares, respectively, show distributions for two representative age ranges: 30–32 and 50–52. The red and blue lines, respectively, show Poisson distributions with means equal to the 30–32 and 50–52 age ranges (i.e., the distributions that would result if partnership numbers were distributed at random). The five panels all show distributions from year 25 after a simulated TasP campaign with *S*_targ_ = 100% (i.e., from simulations with a minimal number of AIDS deaths).
(TIF)

S5 Fig Rollouts in which the number of people being treated increases linearly until everyone has been treated following untargeted (black and blue lines) and “under age 30” (red and green lines) strategies. The left hand, center, and right-hand column gives outcomes following slow, moderate, and fast increases in the number of people being treated over time. The x-and y-axes are same as in Fig 2 in the main text.
(TIF)

S6 Fig Time-discounted AIDS deaths for the experiment shown in Fig 3. The x-axis and other strategies are described in Fig 3. Data in the top-left panel is identical to the top-right panel in Fig 3. Error bars have been left out for clarity.
(TIF)

S7 Fig Performance of strategies that consider both CD4 and age (grey and blue lines) relative to untargeted “random” (black lines) and age-based TasP (brown and red lines). The x- and y-axes and other strategies are described in Fig 3.
(TIF)

S8 Fig Repeat of S7 Fig with a lower CD4 cutoff. The x- and y-axes and other strategies are described in Fig 3.
(TIF)

S9 Fig Time-discounted AIDS deaths for the experiment shown in S8 Fig. The x-axis and other strategies are described in Fig 3. Data in the top-left panel is identical to the top-right panel in S8 Fig. Error bars left out for clarity.
(TIF)

S10 Fig Performance of strategies with sex-specific age targets (blue and purple lines) compared to untargeted “random” (black lines) and age-based TasP with ordinary, sex-independent, age targets (brown, red, and orange lines). The x- and y-axis and other strategies are described in Fig 3.
(TIF)
