# Supplementary material for: Large benefits to youth-focused HIV treatment-as-prevention efforts in generalized heterosexual populations: An agent-based simulation model
Source: PLoS Comput Biol. 2019 Dec 17;15(12):e1007561. doi: 10.1371/journal.pcbi.1007561 (PMC6938382; doi:10.1371/journal.pcbi.1007561)
Supplement: S1 Text — Gives an overview of the Evonet_HIV package. (DOCX) [file pcbi.1007561.s001.docx]

**Supplementary Methods**

Large benefits to youth-focused HIV treatment-as-prevention efforts in generalized heterosexual populations: An agent-based simulation model.

Table of Contents

1. Model overview 2

2. Network Estimation Procedures 2

3. Burn-in period 2

4. Sexual frequency, condom use, and male circumcision 3

5. HIV transmission 3

6. Set point viral load 4

7. Viral dynamics 5

8. Disease progression 6

9. Age distributions 7

9.1 Model initialization 7

9.2 Entries 8

9.3 Deaths 8

9.4 Aging 8

10. Antiretroviral treatment 9

10.1 Effect of ART on viral dynamics 9

10.2 Effect of ART on disease progression 10

11. Testing and diagnosis 10

12. Use of proxies for CD4-based and VL-based targeting strategies 11

13. References 11

# Model overview

*EvoNetHIV* is a stochastic, agent-based simulation model that incorporates sexual network structure, behavior, HIV evolution, and treatment. Each simulation first estimates a statistical model that governs sexual network structure, and then proceeds through a burn-in period and epidemic simulation. At each time step of both the burn-in period and epidemic simulation, (1) partnerships form and dissolve; (2) sexual acts take place within a subset of existing partnerships; (3) HIV transmission occurs probabilistically within a subset of sexual acts; (4) viral dynamics and disease progression are updated for each infected agent; (5) vital dynamics, such as aging, are updated, and (6) testing and treatment are implemented at user-specified intervals. These processes are described in detail below.

*EvoNetHIV* is programmed in the R software language (R Development Core Team, 2008). Model code is accessible at <https://github.com/EvoNetHIV/Mittler-et-al-TasP-by-Age>. *EvoNetHIV* is written as a series of modules, with multiple options for each module and the option to write additional modules. It also includes over 100 parameters that users can modify from default values. Here we describe the *EvoNetHIV* components and parameters used in this paper; for more details, see <https://github.com/EvoNetHIV>. This supplement describes an updated version of the ***Evonet_HIV*** software package that allows users to investigate treatment-as-prevention (TasP) strategies.

This write-up borrows extensively from previous technical supplements for ***Evonet_HIV*** (Goodreau *et al.* 2018, Herbeck *et al.* 2018, and Stansfield *et al.* 2018). Items that are new to this version are highlighted in the dark blue font used here.

Some simulations were conducted on the Hyak supercomputer system at University of Washington, an advanced computational, storage, and networking infrastructure provided by funding through the Student Technology Fee and the Center for Studies in Demography and Ecology.

# Network Estimation Procedures

Networks are estimated with separable temporal exponential random graph models (ERGMs) (Krivitsky and Handcock (2014) as implemented in the statnet (Handcock *et a*l. 2003) and EpiModel (Jenness *et al.* 2016a) suites. These statistical models also simulate a dynamic network that maintains desired network statistics (e.g., mean degree) over model duration. The parameter estimates obtained for the initial network are then used in each subsequent time step of the simulation to update the network configuration. We use the offset method of Krivitsky, *et al.* (2011) to account for the changing size of the network as the simulation progresses.

# Burn-in period

We start the simulations 20 years before the targeted TasP campaign to give time-dependent quantities (e.g., the percentage of couples that are serodiscordant) a chance to equilibrate before modeling the effect of targeted TasP strategies.

# Sexual frequency, condom use, and male circumcision

Sexual acts are determined among agents in a serodiscordant relationship at each time step. Among these partnerships, the number of sexual acts per partnership at a given time step is assigned according to a Poisson draw with mean equal to the inverse of expected number of sex acts per day. Functions describing age-dependent probabilities of sex and condom usage are in the main text. We assume that 40% of male agents are circumcised at time of entry to model.

# HIV transmission

HIV transmission probabilities are calculated as a function of relevant risk factors for each sex act according to characteristics of the sexual act and characteristics of the agents engaged in the sexual act. Following Hughes *et al.* (2012), the probability of transmission is calculated for each sexual act that occurs in a serodiscordant relationship, as:

$$P\left( transmission \right)=1-{(1-\lambda)}^{e^{X\beta}}$$

where

$$X\beta=\ln\left( 2.89 \right)*\left( viral load-4.0 \right)+ ln(1.492) * decades\_under\_46 +\ln\left( 0.22 \right)*condom+\ln\left( 1.5 \right)*female\_recipient+\ln\left( 0.53 \right)*circumcised$$

where $viral load$ is log_10_ of plasma viral load; $decades\_under\_46$ is min{0, (46-*age*)/10}; *age* is the age of the susceptible partner; and *condom*, *female_recipient*, and *circumcised* are binary [0/1] variables that equal 1 if the couple is using a condom, if the seronegative partner is female, and if seronegative partner is a circumcised male, respectively.

# Set point viral load

Set point viral load (SPVL) in infected agents at model initialization is generated as a combination of viral and environmental factors. The viral contribution to SPVL is drawn from a normal distribution with mean 4.5 log_10_ copies/mL and standard deviation of $\sqrt{h^{2}\times variance of {log}_{10} SPVL}$. The environmental contribution is drawn from a normal distribution with mean of 0 and standard deviation of $\sqrt{{(1-h}^{2})\times variance of {log}_{10} SPVL}$. SPVL is then the sum of the viral and environmental contributions, constrained to a minimum value of 2 log_10_ copies/mL and a maximum value of 7 log_10_ copies/mL.

Upon transmission, the SPVL of a newly infected agent is determined by the SPVL of the donor virus, viral mutational variance, and an environmental contribution. The viral mutational variance is drawn from a normal distribution with mean 0 and standard deviation 0.01. The environmental contribution to the SPVL of newly infected agents is drawn from the same distribution as that of infected agents at model initialization. The SPVL of newly infected agents is then the sum of the inherited SPVL of the donor agent, mutational variance, and an environmental contribution.

**Table 5.1**. Parameters determining HIV transmission probability per serodiscordant sexual act

| **Model parameter** | **Value** | **Source(s) and notes** |
| --- | --- | --- |
| Per-act infectivity (λ) | 0.00247 | Increased 15-fold from the value in Hughes *et al*. (2012), ~3-fold from the value in Patel *et al.* (2014). We and others have found it very hard to generate an appreciable epidemic using values from Hughes *et al*. and Patel *et al*. without making some *ad hoc* adjustments. Here created an appreciable epidemic by increasing per-act transmission rates. The latter can be justified on the basis of the uncertainty in infectivity rates documented by Patel *et al*. (2014) |
| Viral load base | 4.0 | J. Hughes, personal communication, November 14, 2014 |
| Relative risk of log_10_ increase in viral load | 2.89 | Hughes, *et al.*, 2012 |
| Relative risk if susceptible partner is female | 1.5 | Patel *et al.* (2014). Hughes *et al.* (2012) reported a relative risk of 1.95 (*P* = 0.003) in a univariate analysis, but only 1.03 (*P* = 0.93) in a multivariate analysis that accounted for men having higher VLs in their dataset. Our use of Patel's value may compensate for males not having higher VLs in our model. |
| Relative risk of condom use | 0.22 | Hughes, *et al.*, 2012 |
| Relative risk if susceptible male is circumcised | 0.53 | Hughes, *et al.*, 2012 |
| Increased per-act risk of infection for each decade under 46 | 1.492 | J. Hughes, personal communication, November 14, 2014. Value applies to susceptible people only. A 16-year old will have a (1.492)^3^ = 3.3-fold higher per-act risk of getting infected than a 46-year old. |

**Table 6.1**. Parameters utilized in the assignment of set point viral load

| **Model parameter** | **Value** | **Source(s) and notes** |
| --- | --- | --- |
| Mean log_10_ SPVL at model initialization | 4.5 | Fraser *et al*. (2007), Korenromp *et al.* (2009) |
| Heritability of SPVL across transmissions (h^2^) | 0.36 | Fraser *et al.* (2014) |
| Variance of log_10_ SPVL | 0.8 | Herbeck *et al.* (2012) |
| Mutational variance | 0.01 | There are no published estimates of mutational variance. We have therefore programmed a low value to be conservative and to maintain approximately 0.36 heritability output measure. |

#

# Viral dynamics

Upon infection, viral load, *V*, grows exponentially at rate *r*_0_ for the first 21 days,

$$V\left( t \right)=V_{0}e^{r_{0}t};t\leq21$$

where *V*_0_ is the initial value (set to 0.0001 copies/mL) and *t* indicates the number of days since initial infection. Using the regression model of Robb *et al.* (2016), peak viral load is a function of the agent’s SPVL,

$$V_{peak}=4.639+0.495*{log}_{10}\left( SPVL \right).$$

where the values of 4.639 and 0.495 are based on regression data given in Robb et al. (2016). We set *r*_0_ = *ln*(*V*_peak_/*V*_0_)/21 in order to obtain peak viral load on day 21. After reaching peak viral load, viral load decays biphasically. The first phase has a duration of 11 days, in which viral load decays linearly :

$$V\left( t \right)=V_{peak}\left( \frac{V_{32}}{V_{peak}} \right)^{\frac{(t-21)}{11}}$$

where viral load at $t$ =32 is a weighted geometric mean of V_adj_peak_ and SPVL:

$$V_{32}={SPVL}^{0.714}*V_{peak}^{0.286}$$

For the remainder of the duration of acute infection, viral load declines linearly until reaching the agent’s SPVL at day 90 of infection. Viral load decay in this phase is

$$V\left( t \right)=V_{32}\left( \frac{SPVL}{V_{32}} \right)^{\frac{(t-32)}{58}}.$$

In the chronic phase of HIV infection, an agent’s viral load increases at a constant annual rate of 0.14 log_e_ copies/mL per year:

$$V\left( t \right)=SPVL*e^{0.14*\frac{t-90}{365}}.$$

This trajectory continues until an agent initiates antiretroviral treatment or enters the AIDS stage, defined by CD4 less than 200 cells/mm^3^. During the AIDS stage, the agent’s viral load increases linearly by 1.004112-fold per day:

$$V(t)=1.004112*V(t-1)$$

Viral load in AIDS increases up to a maximum viral load of 2,400,000 copies/mL.


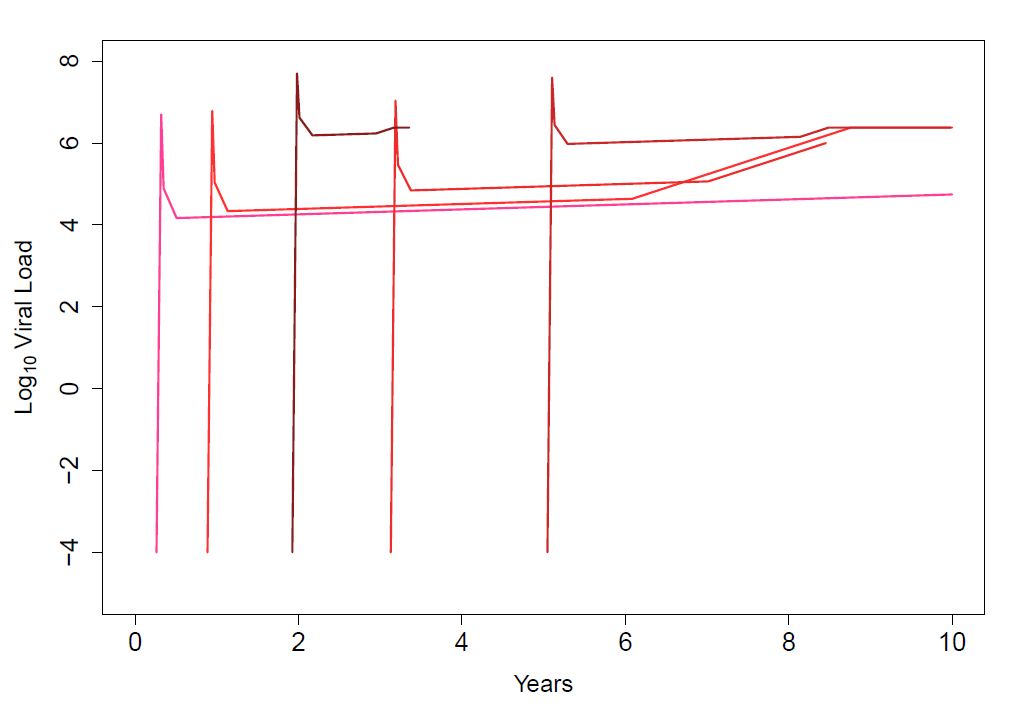


**Figure 7.1**. Viral load dynamics in five agents in an example simulation of ten years.

**Table 7.1**. Parameters utilized in viral load dynamics

| **Model parameter** | **Value** | **Source(s) and notes** |
| --- | --- | --- |
| Viral load at day 0 of infection | 0.0001 | Model-calibrated to replicate viral dynamics in Lindback *et al.* (2000) |
| r_0_ | 1.194 | Model-calibrated to replicate viral dynamics in Lindback *et al.* (2000) |
| Duration of exponential viral growth | 21 days | Lindback *et al.* (2000) |
| Duration of phase 1 decay | 11 days | Lindback *et al.* (2000) |
| Duration of phase 2 decay | 58 days | Lindback *et al.* (2000) |
| Duration of acute infection | 90 days | Fiebig *et al.* (2003) |
| Viral load progression rate, natural log | 0.14 | Geskus *et al.* (2007) |
| Maximum viral load in AIDS (CD4<200) | 2.4x10^6^ copies/mL | Piatak *et al.* (1993) |

# Disease progression

CD4 values determine the additional risk of death among infected agents. Values are categorized as CD4 ≥ 500 cells/mm^3^, 500 < CD4 ≤ 350, 350 < CD4 ≤ 200, and CD4 < 200. Agents are assigned a CD4 category probabilistically at time of infection according to their set point viral load (Cori, *et al.*, 2015; Table 7.1). No agents are assigned a CD4 category of less than 200 cells/mm^3^ upon initial infection.

**Table 8.1**. Probability of assignment to CD4 category stratified by set point viral load

| Set point viral load (log_10_ copies/mL) | CD4 level (cells/mm^3^) | | |
| --- | --- | --- | --- |
|  | ≥ 500 | 350 – 500 | 200 – 350 |
| [2.0, 3.0] | 0.88 | 0.12 | 0.00 |
| (3.0, 3.5] | 0.87 | 0.12 | 0.01 |
| (3.5, 4.0] | 0.85 | 0.12 | 0.03 |
| (4.0, 4.5] | 0.78 | 0.19 | 0.03 |
| (4.5, 5.0] | 0.73 | 0.21 | 0.05 |
| (5.0, 5.5] | 0.71 | 0.25 | 0.04 |
| (5.5, 6.0] | 0.64 | 0.27 | 0.09 |
| (6.0, 6.5] | 0.00 | 0.00 | 1.00 |
| (6.5, 7.0] | 0.00 | 0.00 | 1.00 |

In the absence of antiretroviral treatment, infected agents progress through CD4 categories probabilistically according to a geometric distribution with mean *p*^-1^, where *p* is the inverse of the mean amount of time that an individual remains in a specified CD4 category. The mean duration of time in each CD4 category is determined by SPVL (Cori, *et al.*, 2015 and personal communication; Table 7.2).

**Table 8.2**. Mean time (in years) spent in each CD4 category stratified by set point viral load

| Set point viral load (log_10_ copies/mL) | CD4 level (cells/mm^3^) | | | |
| --- | --- | --- | --- | --- |
|  | ≥ 500 | 350 – 500 | 200 – 350 | < 200 |
| [2.0, 3.0] | 6.08 | 5.01 | 3.60 | 4.67 |
| (3.0, 3.5] | 4.69 | 2.52 | 3.68 | 4.11 |
| (3.5, 4.0] | 3.94 | 4.07 | 2.38 | 3.54 |
| (4.0, 4.5] | 2.96 | 3.09 | 3.81 | 2.98 |
| (4.5, 5.0] | 2.25 | 2.32 | 3.21 | 2.42 |
| (5.0, 5.5] | 1.47 | 1.55 | 2.27 | 1.86 |
| (5.5, 6.0] | 0.95 | 1.19 | 1.00 | 1.29 |
| (6.0, 6.5] | 0.32 | 0.59 | 0.68 | 0.73 |
| (6.5, 7.0] | 0.30 | 0.46 | 0.37 | 0.17 |

For this paper, we added a flag that to account for data given in Cori *et al.* (2015) indicating that CD4 counts decline more rapidly in older HIV+ people. When this flag is set, the progression times given in Table 7.2 are multiplied by the numbers in Table 1 in the main text.

# Age distributions

## 9.1 Model initialization

Initial population size is either 2,000, 10,000, or 20,000 with HIV prevalence of 7.5%. Initial age distribution is calculated with an algorithm that yields a steady-state age distribution in the absence of HIV-induced mortality (Figure 8.1).


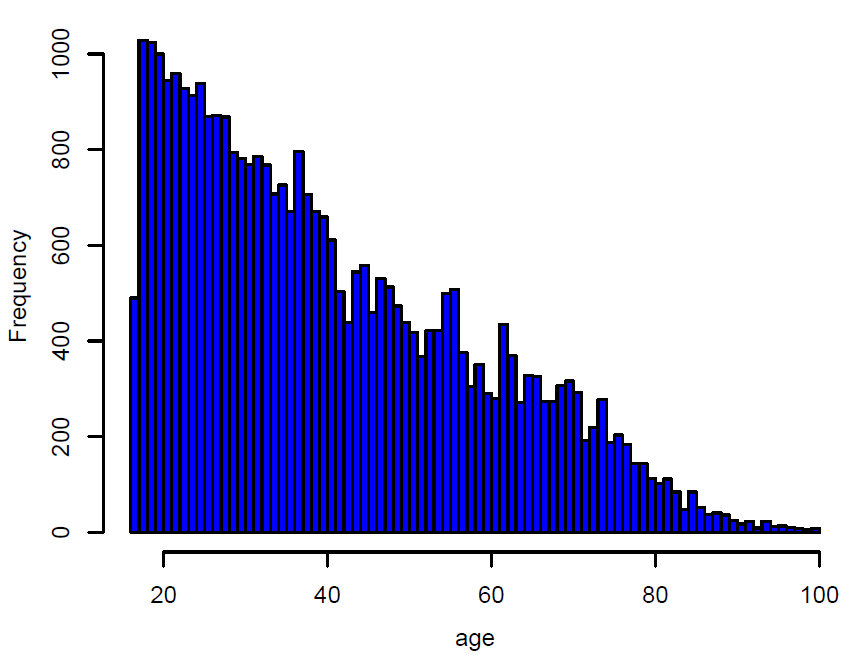


Figure 9.1. Age distributions from a simulation of a successful TasP campaign.

## 9.2 Entries

The model was calibrated to yield a 1% annual population growth in the absence of HIV-induced mortality. Each new agent enters the model uninfected at age 16, with the actual age of entry being a random number between 16 and 17.

## 9.3 Deaths

Age-specific annual mortality rates, subsequently converted to daily probabilities, come from the UW Institute of health metrics (IHME) compilation of data obtained from Global Burden of Disease Study (2013) (downloaded from http://ghdx.healthdata.org/gbd-results-tool in July 2017).

Natural deaths occur according to each agent’s age-specific probability of death. HIV-infected agents with CD4 greater than 200 cells/mm^3^ have an increased probability of death that is dependent on their CD4 category. (Table 8.1)

AIDS deaths occur when an infected agent passes through category 4 according to the disease progression matrix in Section 7. Additionally, HIV+ agents are assumed to have higher natural mortality rates, see Table 8.1.

## 9.4 Aging

Agent age is incremented by 1/365 at each time step.

**Table 9.1**. Vital dynamics parameters

| **Model parameter** | **Value** | **Source(s) and notes** |
| --- | --- | --- |
| Initial population size | 2,000 | NA |
| Initial prevalence | 7.5% | Model assumption |
| Annual population growth rate | 1% | New entrants assumed to be HIV negative |
| Minimum age | 16 | NA |
| Maximum age | 100 | NA |
| Age distribution | Calculated | Set to the stable age-distribution that would results in the absence of AIDS deaths. |
| Age-specific annual mortality rates for females (5-year bins for ages 5-80, one bin for ages 80+) | 0.0013, 0.0035, 0.0072, 0.0113, 0.0130, 0.0129, 0.0127, 0.0125, 0.0127, 0.0194, 0.0269, 0.0379, 0.0563, 0.1403 | Global Burden of Disease Study (2013). Data downloaded from the Institute of Health Metrics http://ghdx.healthdata.org/gbd-results-tool in July 2017 |
| Age-specific annual mortality rates for males (5-year bins for ages 5-80, one bin for ages 80+) | 0.0018, 0.0039, 0.0071, 0.0117, 0.0157, 0.0185, 0.0197, 0.0197, 0.0219, 0.0325, 0.0441, 0.0582, 0.0815, 0.1629 | Global Burden of Disease Study (2013). Data downloaded from the Institute of Health Metrics http://ghdx.healthdata.org/gbd-results-tool in July 2017 |
| Additional probability of death with CD4 > 500 cells/mm^3^ | 0.0000112 / day | The values from CASCADE (2011) are for men with mean age 30. Rates presented here therefore subtract 0.0014, the natural mortality rate for North American males aged 30 (CDC 2015), to estimate an excess death rate associated with this CD4 category. |
| Additional probability of death with CD4 350-500 cells/mm^3^ | 0.0000148 / day | See note above |
| Additional probability of death with CD4 200-350 cells/mm^3^ | 0.0000333 / day | See note above |

# Antiretroviral treatment

## 10.1 Effect of ART on viral dynamics

Following initiation of ART, viral load decays exponentially according to the formula

$$V\left( t \right)=\left\{ \begin{matrix} V\left( t-1 \right)*e^{-0.6}; V\left( t-1 \right)>0.001 \\ V\left( t-1 \right); V\left( t-1 \right)\leq0.001 \end{matrix} \right.$$

where *t* = time in days. The minimal viral load, 0.001 RNA copies/ml, was set well below the one in Palmer *et al.* (2003) to account for the rarity of transmissions from HIV+ persons with suppressed viral loads. With this value, the probability of a suppressed person transmitting is ~8 fold lower than the *upper* bound in Supervie and Breban (2018) – a study that avoided giving a direct estimate due to the extreme rarity of transmissions in the data sets that they analyzed.

**Table 10.1**. Parameters determining antiretroviral treatment and effects

| **Model parameter** | **Default value** | **Range in simulations** | **Source(s) and notes** |
| --- | --- | --- | --- |
| Per day rate of exponential decay of viral load | -0.6 | -0.6 | Ho et al. (1995), Wei, *et al.* (1995), and Perelson *et al.* (1996). |
| Viral load (copies/ml) at suppression | 0.001 | 0.001 | Model assumption. (See text above) |
| Daily probability of improving by one CD4 category when on ART | 0.03 | 0.03 | Pakker *et al.* (1998  ) |
| Daily probability of death among treated individuals with AIDS | 0.000076 | 0.000076 | CASCADE (2011) and Lifson *et al.* (2012) |
| Rebound in viral load after discontinuing therapy | -- | -- | VL increases from suppression level to pre-therapy value 30 days after therapy cessation. |

## 10.2 Effect of ART on disease progression

Following ART initiation, each agent’s CD4 category improves in a memoryless process until reaching CD4 greater than 500 cells/mm^3^. At each time step, a given agent receiving treatment has a 3% probability of improving by one CD4 category. Individuals who initiate treatment while in AIDS stage of infection have an increased daily probability of death (CASCADE 2011; Lifson et al. 2012).

# Testing and diagnosis

Agents have a mean testing interval of one year based on daily testing probability of 1/365. 95% of agents are assumed to test. Agents who have been infected for at least 30 days prior to getting tested will be diagnosed as being HIV positive (Table 10.1). Therapy starts 15 days after testing for eligible agents.

**Table 11.1**. Testing, diagnosis and treatment parameters

| **Model parameter** | **Default value** | **Range in simulations** | **Source(s) and notes** |
| --- | --- | --- | --- |
| Percent of population that tests | 0.95 | 0.5 to 0.95 | Experimental parameter |
| Mean HIV test interval | 1 year | 1 to 3 years | Goodreau, *et al.* (2012) and Jenness, *et al.* (2016b) |
| Minimum possible delay in days between infection and positive HIV diagnosis | 30 | 30 | Approximate value for the average time to being antibody positive. |
| Minimum delay in days between diagnosis and start of therapy | 15 | 15 | Assumes some delay between diagnosis and start of drug regimen. |
| Annual increase in the number treated agents once TasP target reached (*r*) | 2% | 2% | Accounts for population growth and general increases in health care expenditures and productivity |
| Annual probability of dropping out of care | 0 | 0, 0.10 | Set default to 0 to avoid ratcheting targeted agents into therapy. 0.1 is based on Yu *et al.* (2007), Fleishman *et al.* (2013), and Mberi *et al.* (2015). |

# Use of proxies for CD4-based and VL-based targeting strategies

In simulations in which patients discontinue therapy, viral loads will increase and CD4 counts will decrease. Targeting based on VL or CD4 counts, therefore, can induce oscillations as people who have dropped out of care get relinked to care as soon as their viral load rises or CD4 count drops. To avoid such oscillations, we instead use closely related attributes that do not change with treatment, namely SPVL and CD4 nadir (lowest CD4 count observed in a patient). In the absence of dropouts and relinkage to care, the effects of prioritizing agents based on SPVL and CD4 nadir are similar to the effects of prioritizing based on VL and CD4 count, respectively (data not shown).

# References

CASCADE Collaboration (2011) Timing of HAART Initiation and Clinical Outcomes in Human Immunodeficiency Virus Type 1 Seroconverters. *Archives of Internal Medicine* **171**:1560-1569.

Cori *et al.* (2015) CD4(+) cell dynamics in untreated HIV-1 infection: overall rates, and effects of age, viral load, sex and calendar time. *AIDS* **29**:2435-2446.

Fiebig *et al.* (2003) Dynamics of HIV viremia and antibody seroconversion in plasma donors: implications for diagnosis and staging of primary HIV infection. *AIDS* **17**: 871-1879.

Fleishman JA, Yehia BR, Moore RD, Korthuis PT, Gebo KA. (2012) Establishment, retention, and loss to follow-up in outpatient HIV care. *J Acquir Immune Defic Syndr.* **60**:249–259.

Fraser *et al.* (2007) Variation in HIV-1 set-point viral load: Epidemiological analysis and an evolutionary hypothesis. *Proceedings of the National Academy of Sciences of the United States of America* **104**:17441-17446.

Fraser *et al.*, (2014) Virulence and Pathogenesis of HIV-1 Infection: An Evolutionary Perspective. *Science* **343**:1328.

Geskus *et al.*, (2007) The HIV RNA setpoint theory revisited. *Retrovirology* **4**:65.

Global Burden of Disease Study (GBD 2013) (2014) Age-Sex Specific All-Cause and Cause-SpecifiMortality 1990-2013. Seattle, United States: Institute for Health Metrics and Evaluation (IHME).

Goodreau *et al.* (2012) What drives the US and Peruvian HIV epidemics in men who have sex with men (MSM)? *PLoS ONE* **7**, e50522.

Goodreau SM, Stansfield SE, Murphy JT, Peebles KC, Gottlieb GS, Abernethy NF, Herbeck JT, Mittler JE. (2018) Relational concurrency, stages of infection, and the evolution of HIV set point viral load. *Virus Evolution* [In Press]

Handcock *et al.* (2003) Statnet: Software tools for the Statistical Modeling of Network Data.

Herbeck *et al.* (2012) Is the virulence of HIV changing? A meta-analysis of trends in prognostic markers of HIV disease progression and transmission. *AIDS* **26**:193-205.

Herbeck JT, Peebles K, Edlefsen PT, Rolland M, Murphy JT, Gottlieb GS, Abernethy N, Mullins JI, Mittler JE, Goodreau SM. (2018) HIV population-level adaptation can rapidly diminish the impact of a partially effective vaccine. *Vaccine* **36**:514-520.

Ho *et al.* (1995) HIV-1 dynamics in vivo. *Journal of Biological Regulators and Homeostatic Agents* **9**: 76-77.

Hughes JP, Baeten JM, Lingappa JR, Magaret AS, Wald A, de Bruyn G, Kiarie J, Inambao M, Kilembe W, Farquhar C, Celum C. (2012) Partners in Prevention HSV/HIV Transmission Study Team. Determinants of per-coital-act HIV-1 infectivity among African HIV-1-serodiscordant couples. *J Infect Dis*. **205**:358-365.

Jenness *et al.* (2016a) EpiModel: Mathematical Modeling of Infectious Disease.

Jenness *et al.* (2016b) Impact of the Centers for Disease Control's HIV Preexposure Prophylaxis Guidelines for Men Who Have Sex With Men in the United States. *J Infect Dis* **214**:1800-1807

Korenromp *et al.* (2009), Clinical Prognostic Value of RNA Viral Load and CD4 Cell Counts during Untreated HIV-1 Infection-A Quantitative Review. *PLoS ONE* **4**, e5950

Krivitsky and Handcock (2014) A separable model for dynamic networks*. Journal of the Royal Statistical Society Series B-Statistical Methodology* **76**:29–46.

Krivitsky *et al.* (2011) Adjusting for network size and composition effects in exponential-family random graph models. *Statistical Methodology* **8**:319-339.

Lifson *et al.* (2012) Clinical, demographic and laboratory paramters at HAART initiation associated with decreased post-HAART survival in a U.S. military prospective HIV cohort. *AIDS Research and Therapy* **9**:4.

Lindback *et al.* (2000), Viral dynamics in primary HIV-1 infection. AIDS 14, 2283-2291.

Mberi MN, Kuonza LR, Dube NM, Nattey C, Manda S, Summers R. (2015) Determinants of loss to follow-up in patients on antiretroviral treatment, South Africa, 2004-2012: a cohort study. *BMC Health Serv Res*. **15**:259.

Nguyen, et al., 2015. Sexual partnership patterns among South African adolescent girls enrolled in STI Preventions Trial Network 068: Measurement challenges and implications for HIV/STI transmission. *Sexually Transmitted Diseases* **42**:612-618.

Pakker *et al.* (1998) Biphasic kinetics of peripheral blood T cells after triple combination therapy in HIV-1 infection: A composite of redistribution and proliferation. Nature Medicine 4, 208-214.

Palmer *et al.* (2003) New real-time reverse transcriptase-initiated PCR assay with single-copy sensitivity for human immunodeficiency virus type 1 RNA in plasma. *Journal of Clinical Microbiology* **41**:4531-4536.

Patel P, Borkowf CB, Brooks JT. *et al.* (2014) Estimating per-act HIV transmission risk: a systematic review. *AIDS* **28**:1509-1519.

Perelson *et al.* (1996) HIV-1 dynamics in vivo: Virion clearance rate, infected cell life-span, and viral generation time. *Science* **271**:1582-1586.

Piatak *et al.* (1993) High levels of HIV-1 in plasma during all stages of infection determined by competitive PCR. *Science* **259**:1749-1754.

R Development Core Team (2008) R: A language and environment for statistical computing.

Robb *et al.* (2016) Prospective Study of Acute HIV-1 Infection in Adults in East Africa and Thailand. *New England Journal of Medicine* **374**:2120-2130.

Stansfield SE, Mittler JE, Gottlieb GS, Murphy JT, Hamilton DT, Detels R, Wolinsky SM, Jacobson LP, Margolick JB, Rinaldo CR, Herbeck JT, Goodreau SM. (2018) Sexual role and HIV-1 set point viral load among men who have sex with men. *Epidemics* S1755-4365(18)30001-X. [Epub ahead of print]

Wei *et al.* (1995), Viral dyamics in human immunodeficiency virus type-1 infection. *Nature* **373**:117-122.

Yu JK, Chen SC, Wang KY, et al. 2007. True outcomes for patients on antiretroviral therapy who are “lost to follow-up” in Malawi. *Bull. World Health Organ*. **85**: 550–554.
