# Supplementary figures and images for: Large benefits to youth-focused HIV treatment-as-prevention efforts in generalized heterosexual populations: An agent-based simulation model
Source: PLoS Comput Biol. 2019 Dec 17;15(12):e1007561. doi: 10.1371/journal.pcbi.1007561 (PMC6938382; doi:10.1371/journal.pcbi.1007561)

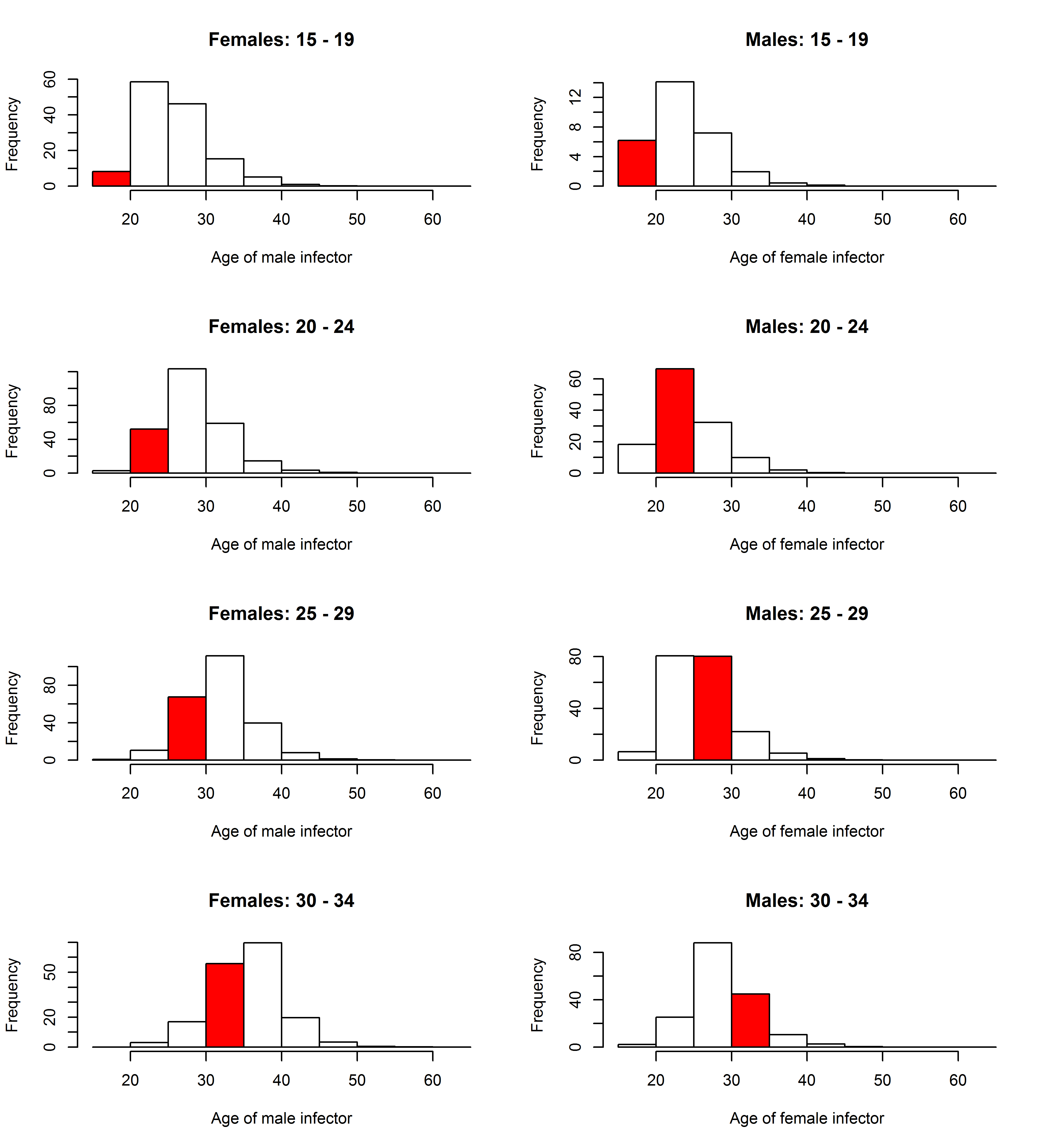

Supplement: S1 Fig — Each histogram shows the ages of the infectors (i.e., the ages of people who infected someone in the group shown in each histogram) in the absence of treatment. The bars show all infection events between years 0 and 5 involving infectors and recipients who were in the relevant age range at the time of infection. Each bar gives the average of 16 replicates. Red bars indicate infectors who were in the same age range as the recipients. (TIF) [file pcbi.1007561.s002.tif]

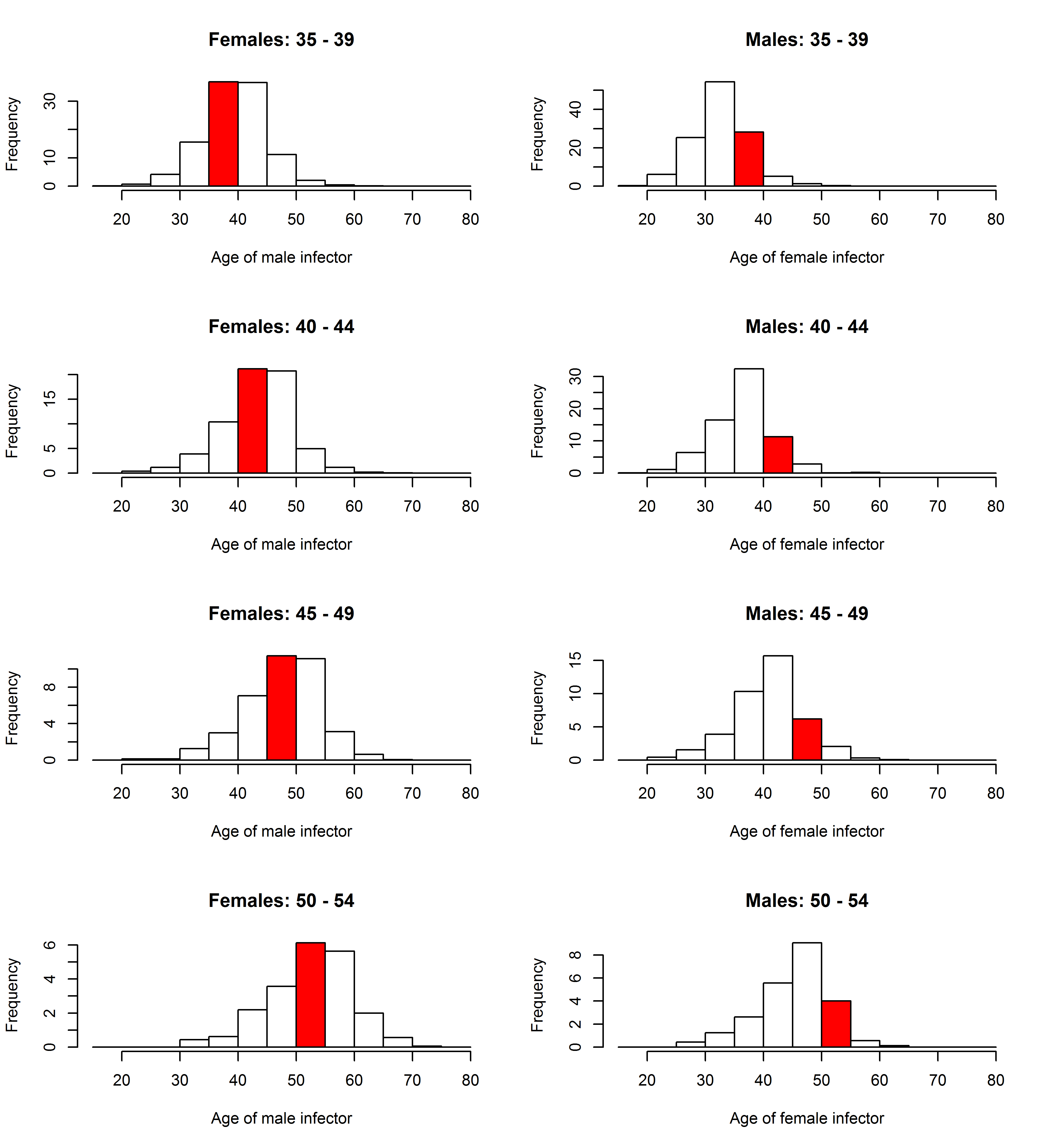

Supplement: S2 Fig — This is a continuation of S1 Fig. Axes, colors, and conditions are described in S1 Fig. (TIF) [file pcbi.1007561.s003.tif]

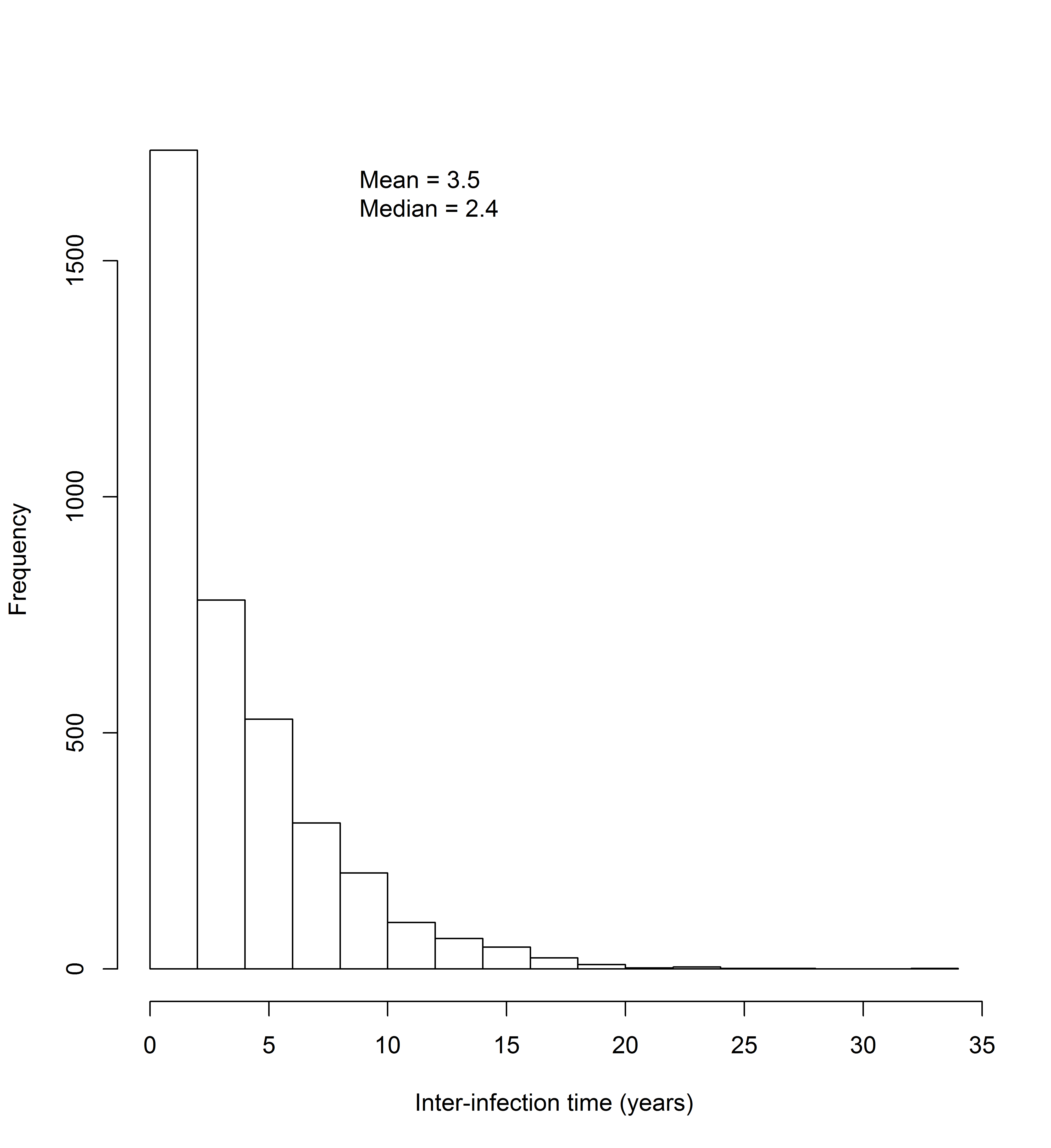

Supplement: S3 Fig — This graph shows the time elapsed between the time of infection and the time that their infector got infected during the last 25 years of a 45-year simulation without treatment. (TIF) [file pcbi.1007561.s004.tif]

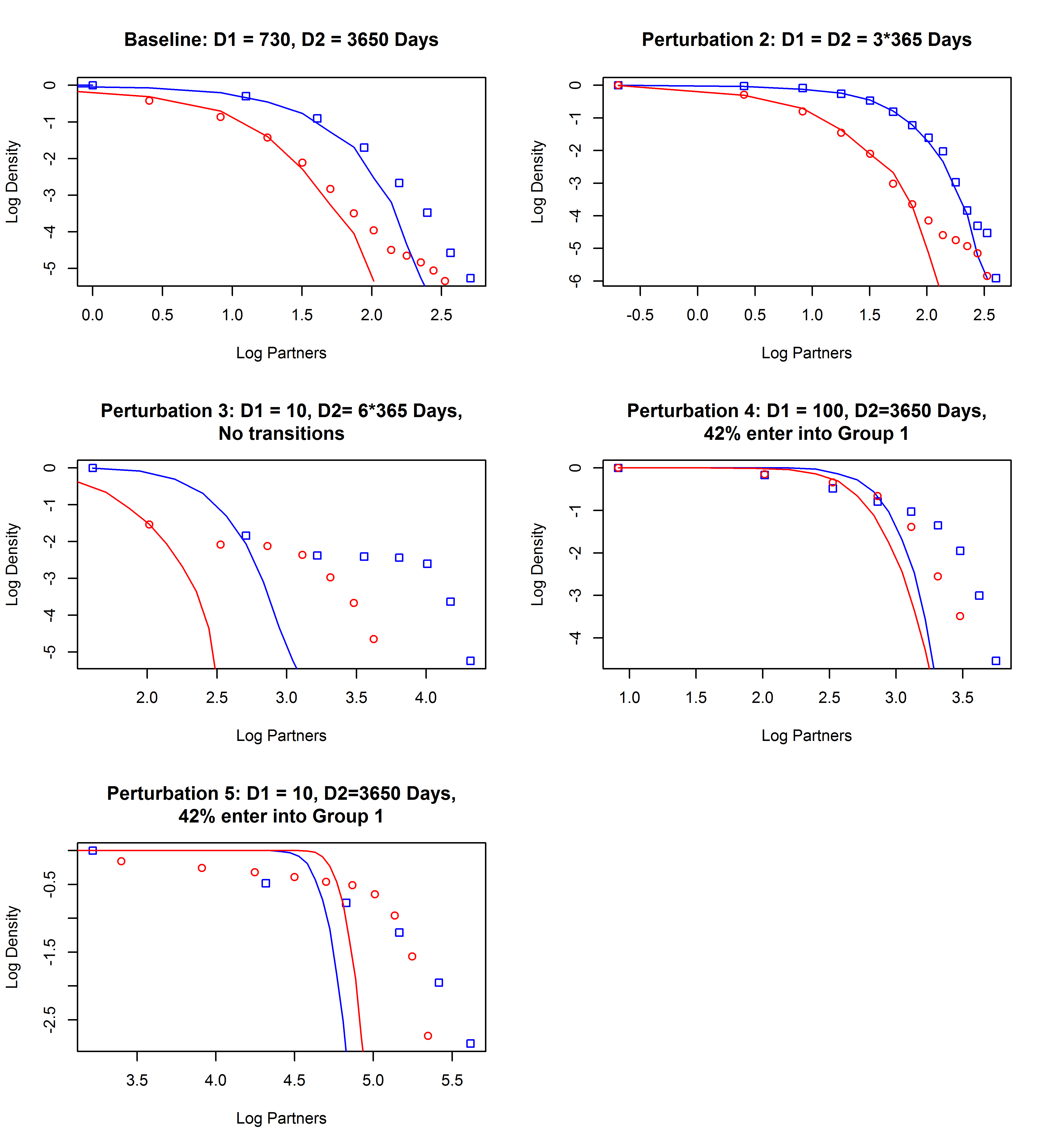

Supplement: S4 Fig — The x-axes give natural logarithm of the total number of partners per person for people. The y-axes give the natural logarithms of cumulative distribution starting with the agent with the most partners. To reduce variation caused by young people not having as much time to form relationships as older people, we plotted distributions for people within narrow age ranges. The red circles and blue squares, respectively, show distributions for two representative age ranges: 30–32 and 50–52. The red and blue lines, respectively, show Poisson distributions with means equal to the 30–32 and 50–52 age ranges (i.e., the distributions that would result if partnership numbers were distributed at random). The five panels all show distributions from year 25 after a simulated TasP campaign with Starg = 100% (i.e., from simulations with a minimal number of AIDS deaths). (TIF) [file pcbi.1007561.s005.tif]

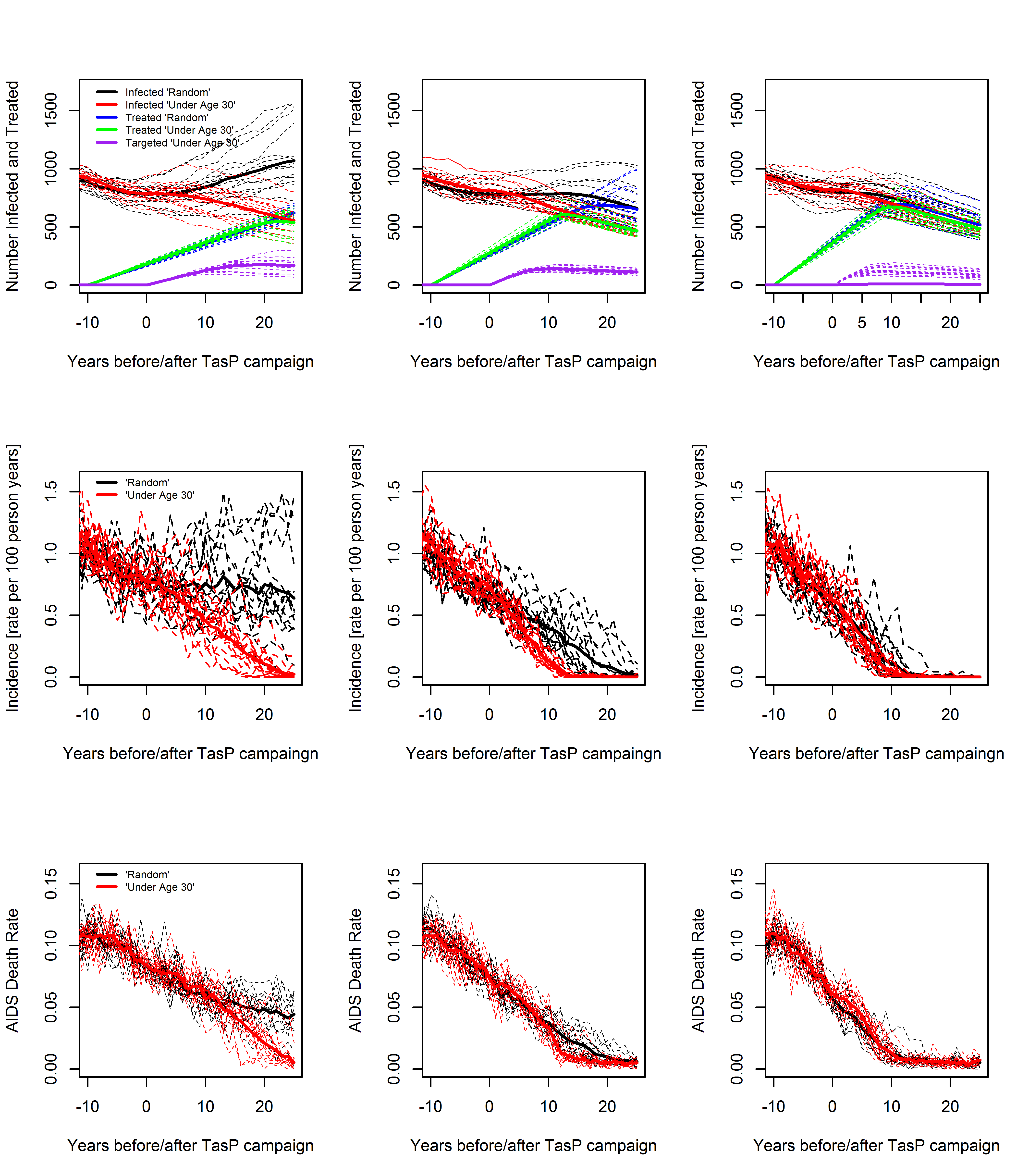

Supplement: S5 Fig — The left hand, center, and right-hand column gives outcomes following slow, moderate, and fast increases in the number of people being treated over time. The x-and y-axes are same as in Fig 2 in the main text. (TIF) [file pcbi.1007561.s006.tif]

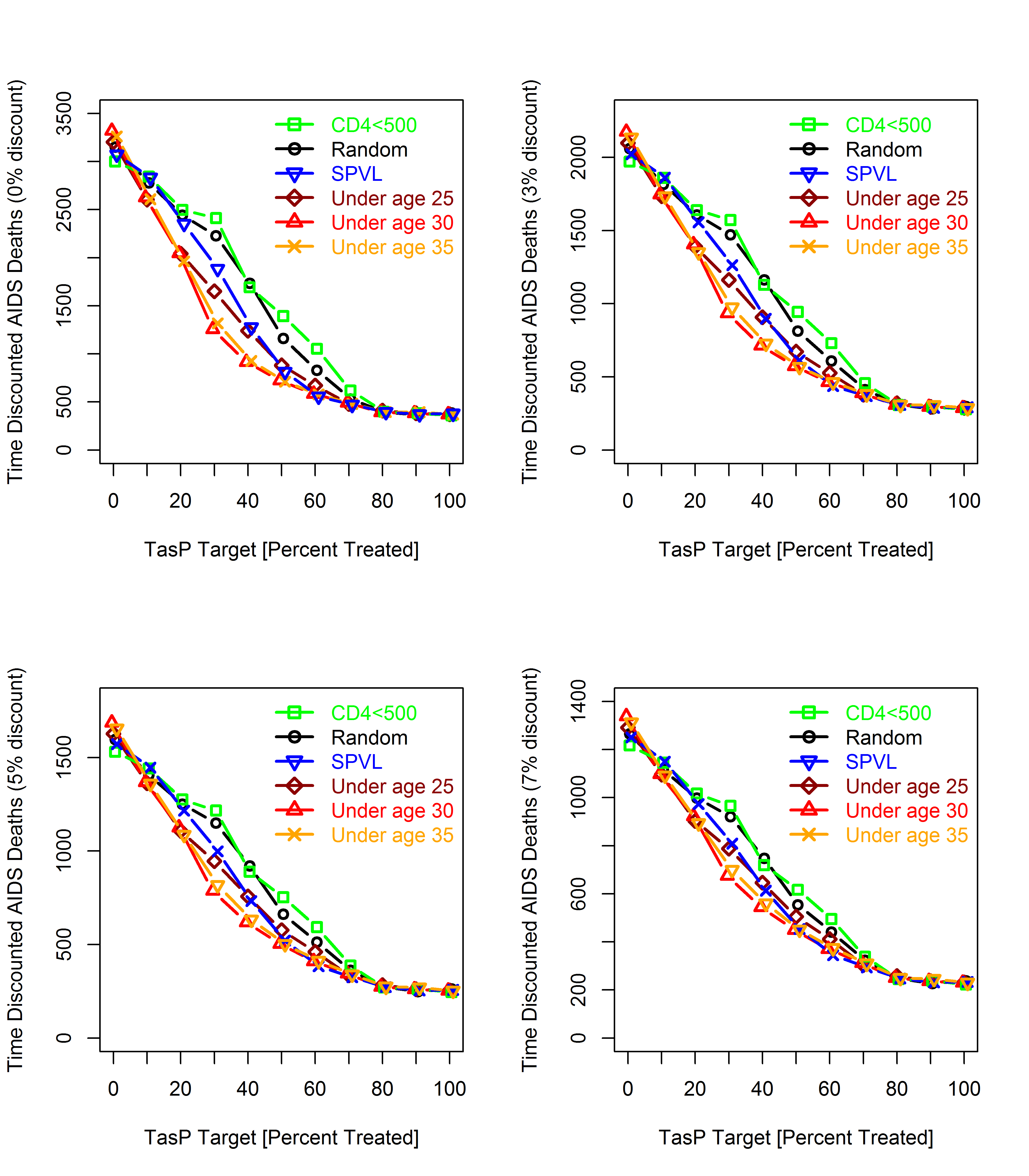

Supplement: S6 Fig — The x-axis and other strategies are described in Fig 3. Data in the top-left panel is identical to the top-right panel in Fig 3. Error bars have been left out for clarity. (TIF) [file pcbi.1007561.s007.tif]

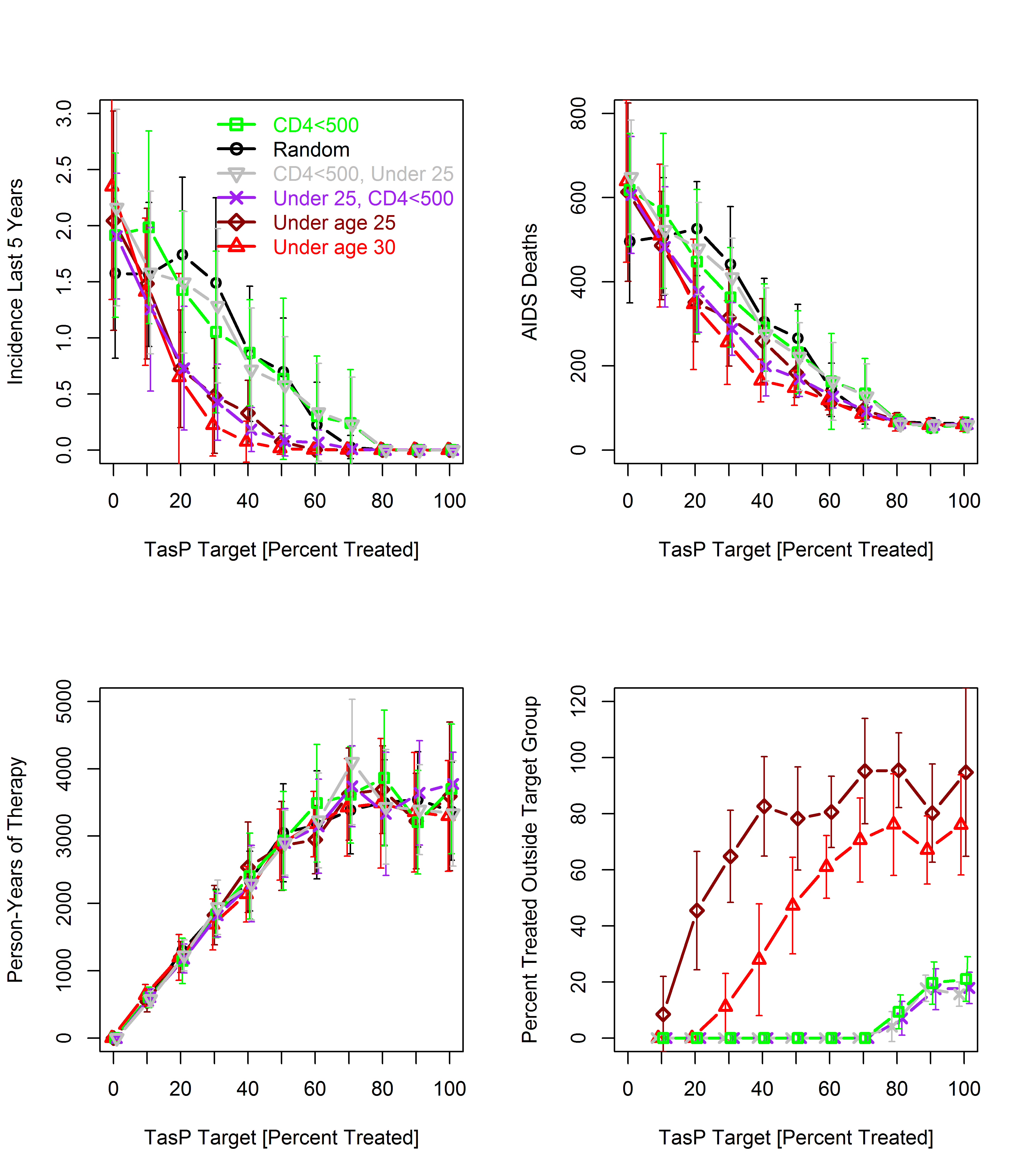

Supplement: S7 Fig — The x- and y-axes and other strategies are described in Fig 3. (TIF) [file pcbi.1007561.s008.tif]

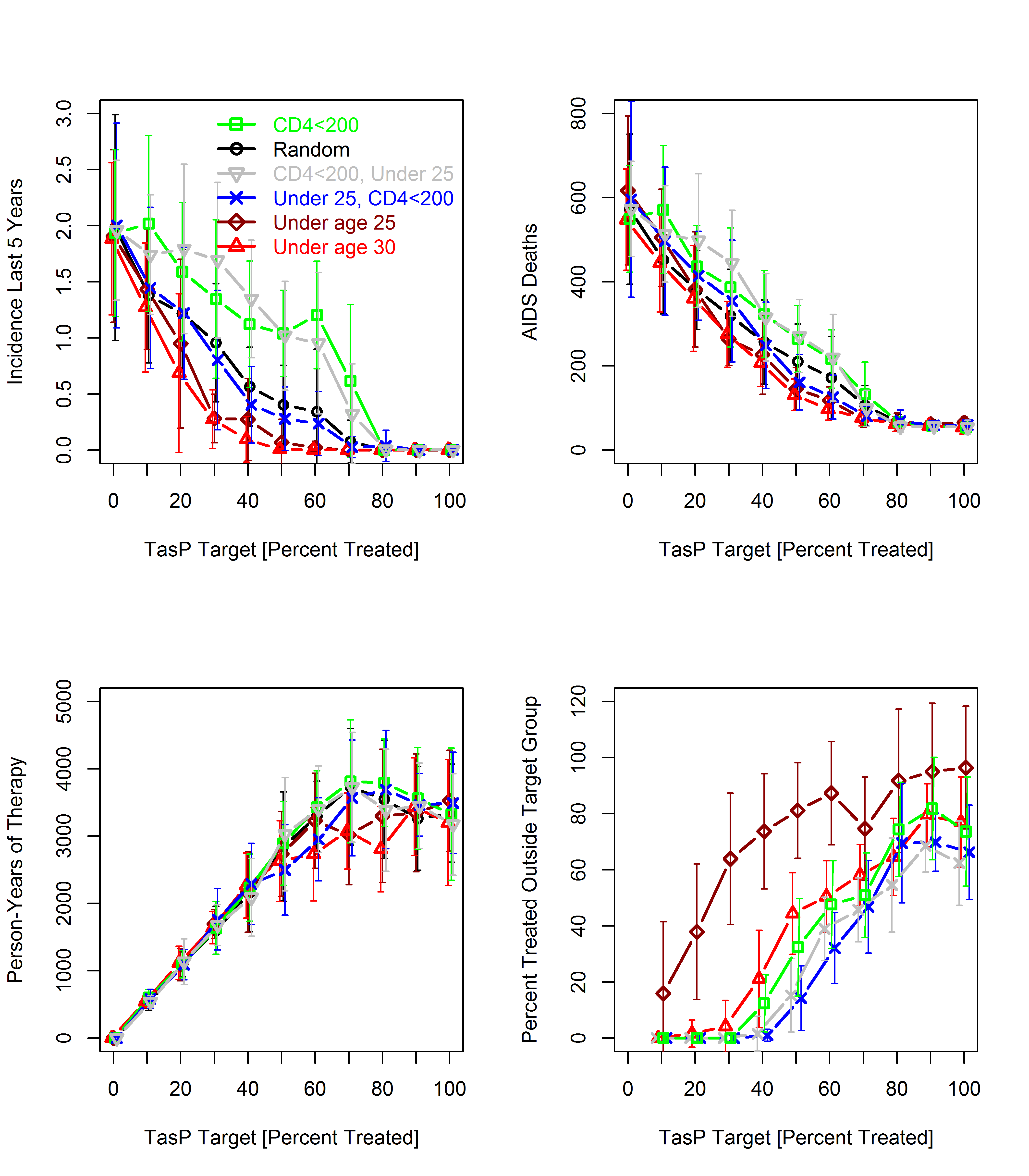

Supplement: S8 Fig — The x- and y-axes and other strategies are described in Fig 3. (TIF) [file pcbi.1007561.s009.tif]

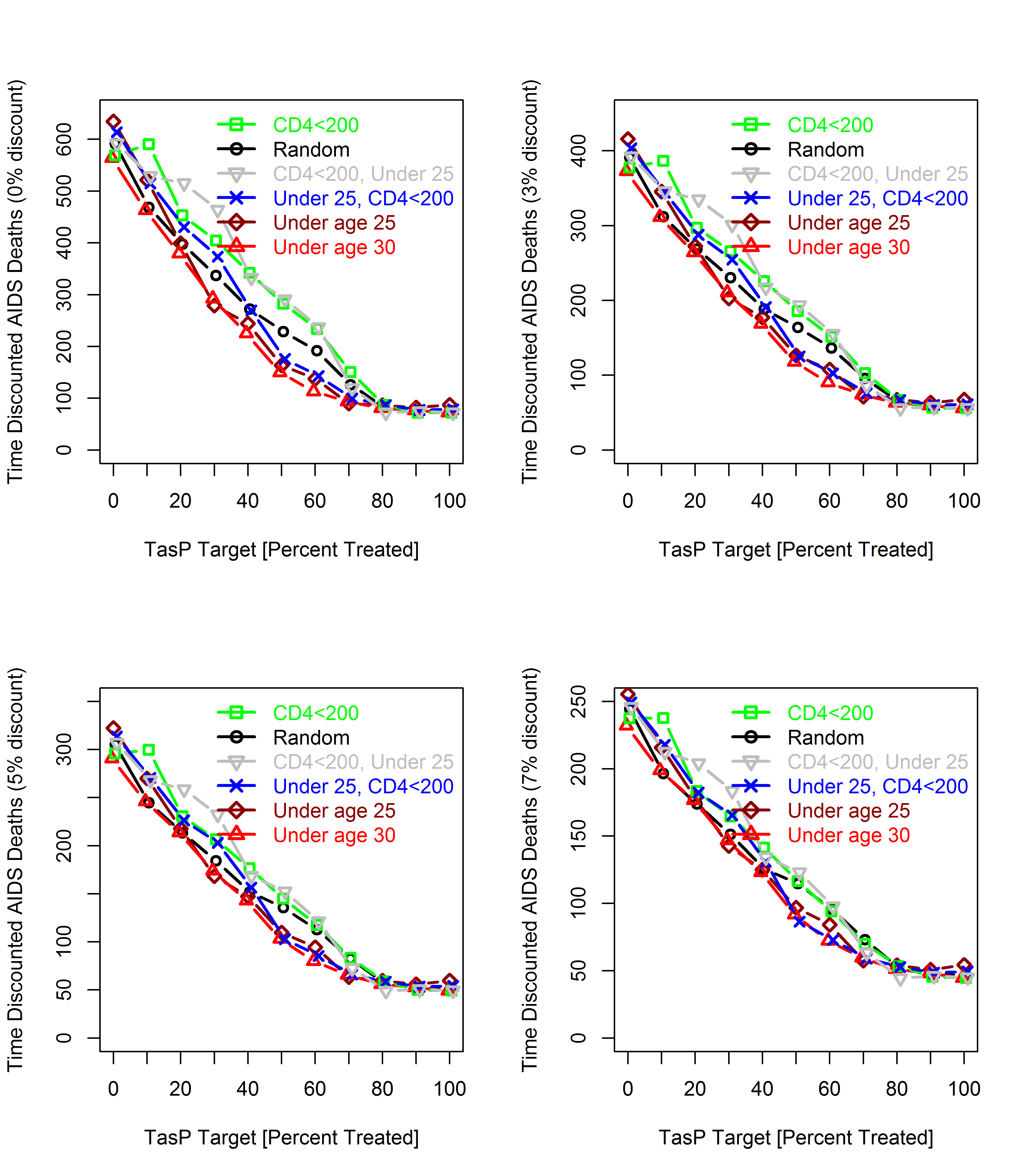

Supplement: S9 Fig — The x-axis and other strategies are described in Fig 3. Data in the top-left panel is identical to the top-right panel in S8 Fig. Error bars left out for clarity. (TIF) [file pcbi.1007561.s010.tif]

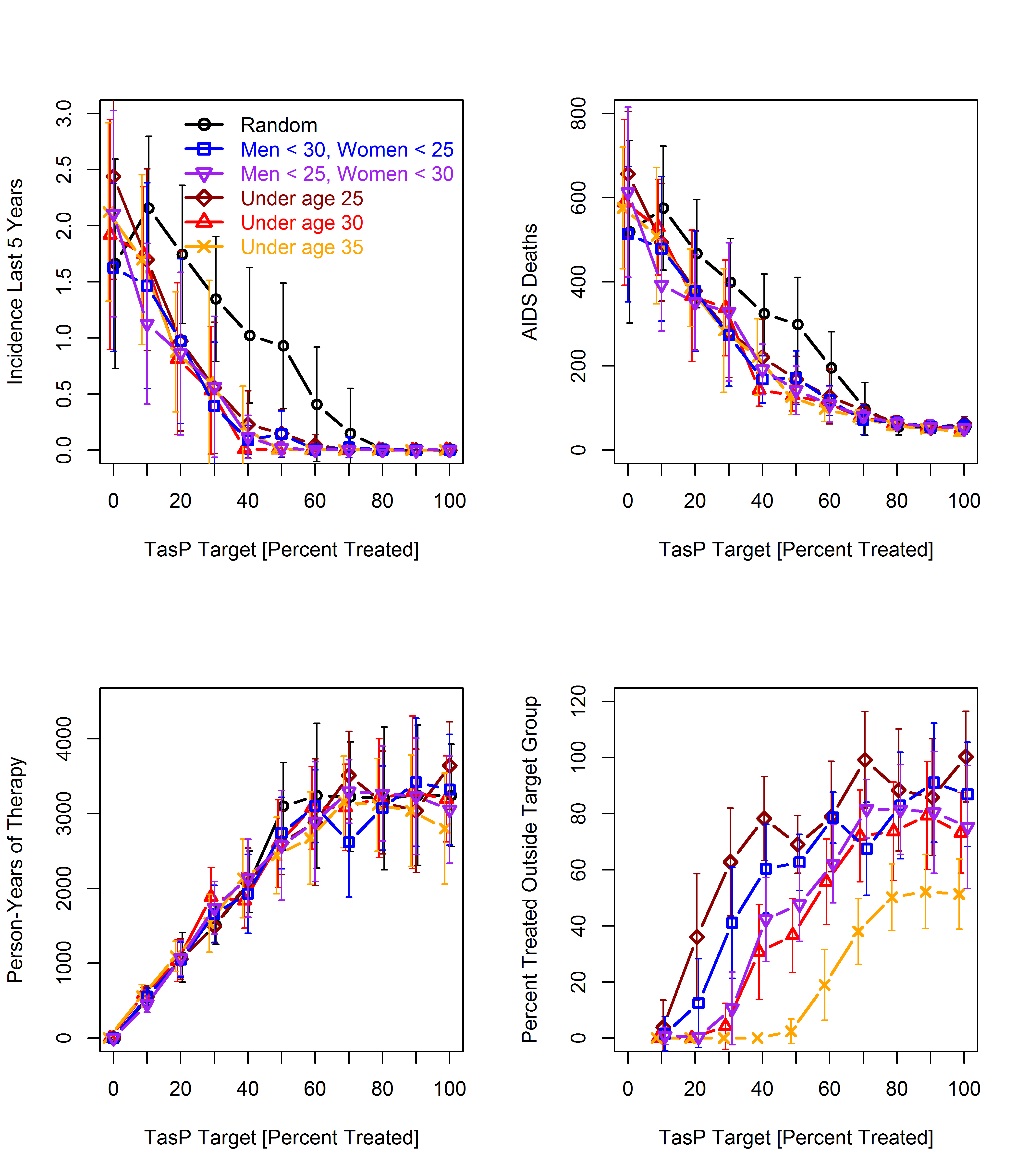

Supplement: S10 Fig — The x- and y-axis and other strategies are described in Fig 3. (TIF) [file pcbi.1007561.s011.tif]
